# Ecological interactions and the underlying mechanism of anammox and denitrification across the anammox enrichment with eutrophic lake sediments

**DOI:** 10.1186/s40168-023-01532-y

**Published:** 2023-04-20

**Authors:** Dandan Zhang, Huang Yu, Yuchun Yang, Fei Liu, Mingyue Li, Jie Huang, Yuhe Yu, Cheng Wang, Feng Jiang, Zhili He, Qingyun Yan

**Affiliations:** 1 Environmental Microbiomics Research Center, School of Environmental Science and Engineering, School of Ecology, Southern Marine Science and Engineering Guangdong Laboratory (Zhuhai), State Key Laboratory for Biocontrol, Sun Yat-Sen University, Guangzhou, 510006 China; 2 Key Laboratory of Aquatic Biodiversity and Conservation of Chinese Academy of Sciences, Institute of Hydrobiology, Chinese Academy of Sciences, Wuhan, 430072 China

**Keywords:** Anammox enrichment, Denitrification, Microbial interactions, Lake, Metagenome sequencing

## Abstract

**Background:**

Increasing attention has recently been devoted to the anaerobic ammonium oxidation (anammox) in eutrophic lakes due to its potential key functions in nitrogen (N) removal for eutrophication control. However, successful enrichment of anammox bacteria from lake sediments is still challenging, partly due to the ecological interactions between anammox and denitrifying bacteria across such enrichment with lake sediments remain unclear.

**Results:**

This study thus designed to fill such knowledge gaps using bioreactors to enrich anammox bacteria with eutrophic lake sediments for more than 365 days. We continuously monitored the influent and effluent water, measured the anammox and denitrification efficiencies, quantified the anammox and denitrifying bacteria, as well as the related N cycling genes. We found that the maximum removal efficiencies of NH_4_^+^ and NO_2_^−^ reached up to 85.92% and 95.34%, respectively. Accordingly, the diversity of anammox and denitrifying bacteria decreased significantly across the enrichment, and the relative dominant anammox (e.g., *Candidatus* Jettenia) and denitrifying bacteria (e.g., *Thauera, Afipia*) shifted considerably. The ecological cooperation between anammox and denitrifying bacteria tended to increase the microbial community stability, indicating a potential coupling between anammox and denitrifying bacteria. Moreover, the *nirS*-type denitrifiers showed stronger coupling with anammox bacteria than that of *nirK*-type denitrifiers during the enrichment. Functional potentials as depicted by metagenome sequencing confirmed the ecological interactions between anammox and denitrification. Metagenome-assembled genomes-based ecological model indicated that the most dominant denitrifiers could provide various materials such as amino acid, cofactors, and vitamin for anammox bacteria. Cross-feeding in anammox and denitrifying bacteria highlights the importance of microbial interactions for increasing the anammox N removal in eutrophic lakes.

**Conclusions:**

This study greatly expands our understanding of cooperation mechanisms among anammox and denitrifying bacteria during the anammox enrichment with eutrophic lake sediments, which sheds new insights into N removal for controlling lake eutrophication.

Video Abstract

**Supplementary Information:**

The online version contains supplementary material available at 10.1186/s40168-023-01532-y.

## Introduction

Nitrogen (N) is a major nutrient that supporting primary productivity of aquatic ecosystems. However, excess reactive N from fertilizer use, sewage discharge, and atmospheric deposition may destroy the ecological balance of the N cycle and result in lake eutrophication [1]. Recently, the anaerobic ammonium oxidation (anammox) was also found to be important for N removal in eutrophic lakes. For example, 13–40% of the nitrogen gas (N_2_) production in lake ecosystems could be contributed by anammox [2, 3]. Anammox bacteria are chemoautotrophs that use free energy released from oxidation–reduction reactions with ammonium (NH_4_^+^) and nitrite (NO_2_^−^) [4, 5]. The anammox bacteria are widely distributed in various engineered and natural ecosystems, such as bioreactors, wastewater treatment plants, ponds, ditches, oceans and groundwater [6–9]. Although the roles of anammox in N removal during wastewater treatments were clearly clarified and widely used [10], much less is known about the underlying mechanism of anammox process in eutrophic lake ecosystems. So far, seven anammox genera (i.e., *Candidatus* Scalindua, *Candidatus* Brocadia, *Candidatus* Kuenenia, *Candidatus* Jettenia, *Candidatus* Anammoxoglobus, *Candidatus* Anammoximicrobium, and *Candidatus* Brasilis) affiliated to the phylum Planctomycetes have been identified [11–13]. Extensive analysis of the anammox gene sequences deposited in the public databases revealed distinct ecological niche differentiations of anammox bacteria in both natural and engineered ecosystems [14]. *Candidatus* Brocadia and *Candidatus* Kuenenia were generally the abundant anammox genera retrieved from natural and engineered ecosystems, respectively. Also, *Candidatus* Jettenia was mainly retrieved from engineered ecosystems, which could proliferate at low N loading rates. Salinity was an important factor governing the distribution of *Candidatus* Scalindua [14]. However, most of enrichments and applications of anammox focused on engineered ecosystems, especially the wastewater treatment plants [15]. Currently, the interpretation of anammox in natural lake ecosystems mainly refer to findings obtained from wastewater studies [16]. The really targeted understanding of anammox bacteria in lakes is mainly restricted to field investigations, with only a few reports about the enrichment of lake anammox bacteria [17, 18]. However, the anammox bacteria and environments in lakes are considerably different from those of wastewater ecosystems.

All anammox bacteria always have a long growth cycle [19] even given with sufficient substrates (NH_4_^+^ and NO_2_^−^) in engineered bioreactors, and the populations are generally shift frequently during the long-term enrichment using bioreactors [4]. In natural ecosystems, microbial metabolites and necromass are the major carbon sources for denitrification [7, 20, 21]. Denitrification and anammox are the major microbial processes that remove the excess N from aquatic ecosystems. Denitrification reduces NO_3_^−^ to NO_2_^−^, NO, N_2_O, and ultimately N_2_ step by step under anaerobic conditions [22]. However, anammox oxidizes NH_4_^+^ with NO_2_^−^ to form N_2_ gas under anaerobic autotrophic conditions. Dissimilatory NO_3_^−^ reduction to ammonium (DNRA) leads to N retention as NH_4_^+^, promoting N recycling. DNRA competes with anammox for NH_4_^+^. Denitrification and anammox are responsible for NO_2_^−^ reduction [23]. At the same time, based on the average potential rates of N loss, the annual N loss rates of denitrification were much higher than that of DNRA [24]. The environmental requirements for anammox and denitrification are similar, but denitrifying bacteria are generally growing better than anammox bacteria under the N and carbon-constrained environments [25]. Although the first anammox enrichment was found in a denitrifying bed [24], an excessive growth of denitrifying bacteria would inhibit the efficiency of anammox enrichment due to their competition for substrate NO_2_^−^ [26]. The anammox and denitrifying bacteria may also compete for some other substrates such as organic carbons [25]. Generally, denitrifying bacteria prefer to use organic matter as carbon sources [25], but the anammox bacteria only compete the organic carbons as electron donors [27]. Some anammox bacteria could oxidize short chain fatty acids, which was coupled with reduction of NO_3_^−^ and/or NO_2_^−^ to NH_4_^+^, disguised as denitrifiers alternatively [28]. However, the abundance of anammox bacteria and their N removal contribution in lake sediments were much lower than that of denitrification. Study efforts have rarely focused on the coupling mechanism of anammox and denitrification in lake ecosystems, which is especially important for determining suitable conditions for the start-up of anammox enrichment with lake sediments [15]. Thus, understanding the ecological interactions and the underlying mechanism of anammox and denitrification are of great importance to establish a rapid start-up of anammox enrichment for studying N removal in eutrophic lakes.

This study aims to explore possible N removal mechanisms during the anammox enrichment with eutrophic lake sediments by focusing on the interactions and coupling between anammox and denitrification. We hypothesized that the ecological interactions, especially through niche differentiation and cooperation between anammox and denitrification would trigger the rapid start-up of anammox enrichment with lake sediments. To test this core hypothesis, we set up four bioreactors (as 4 replicates) to enrich anammox bacteria with eutrophic lake sediments, which collected from lake areas with different trophic levels. The anammox and denitrifying bacteria and N cycling genes were quantified over an enrichment of more than 1 year (i.e., 371 days). Sediments samples were taken at the end of each phase and analyzed using sequencing of 16S rRNA, *nirS/K*, and *hzsB* genes, as well as metagenome sequencing. The interactions of denitrifying and anammox bacteria were analyzed to explore their coupling mechanisms in N removal. Overall, this study expands our understanding of the cooperated N removal by anammox and denitrifying bacteria during anammox enrichment with lake sediments, thus sheds new insights into N regulation for eutrophication control in lake ecosystems.

## Materials and methods

### Experimental design of anammox enrichment in bioreactors with lake sediments

To explore the ecological interactions and the underlying mechanism of anammox and denitrification in lake ecosystems, anammox enrichment with lake sediments was performed in bioreactors. Each bioreactor (5 L) was minorly modified from anaerobic fermentation tank produced by DOE Biotechnology Co., Ltd. (Suzhou, China). Specifically, an inlet and outlet were added at the top of bioreactor to exchange influent and effluent water via a pump (Figure S1). Attachment was enhanced by using polyurethane sponge fillers as microbial carriers. To maintain anoxic conditions, the bioreactors were continuously flushed with argon gas for 30 min at a flow rate of 0.5 LPM (liter per minute) before every test, and tin foil paper was used to cover the bioreactors to block out light. The flow of water was circulated clockwise at 60 r/min to help microorganisms contact each other effectively.

Four anaerobic bioreactors (as 4 replicates) were used to perform the anammox enrichment with surface sediments (0–20 cm), which were collected from four sites that represent different lake areas of this shallow eutrophic lake (Lake Donghu, Wuhan, China) on October 22, 2020 (Table S1). However, we found no significant differences in the microbial community structure among these four sites [29]. At each sampling site, gravity sampling was used to collect surface sediments, which were immediately filled in anaerobic bottles and transported to the laboratory at room temperature, and then placed into each of the four bioreactors (Figure S1). Each bioreactor was seeded with 1.0 kg lake sediments collected from one site of Lake Donghu. There was 3.0 g per liter of volatile suspended solid (VSS) in the seed sludge. The natural environment in lake should be very complex and changeable, we thus provide unique conditions that can be maintained in the natural environment. To enrich anammox bacteria rapidly, the influent concentrations of NH_4_^+^, NO_2_^−^ (Table S2) and temperature were referred to a previous study that enriched the anammox bacteria successfully [16] and lake environments (Table S1 and S2). The reactor was kept at 34 ± 1 °C and a hydraulic retention time (HRT) was set at 48 h and 24 h according to the removal effect. The inorganic compounds including CaCl_2_·2H_2_O (0.135 g/L), KH_2_PO_4_ (0.027 g/L), FeSO_4_·7H_2_O (9.0 mg/L), and MgCl_2_ (0.26 g/L) were also added in the influent water according to [30]. Additional NaHCO_3_ was added to maintain a pH between 7.4 and 7.8.

### Chemical analysis

Ion chromatography meter (ICS-600, Thermo, USA) was used to measure NH_4_^+^, NO_2_^−^ and NO_3_^−^ concentrations. The denitrification and anammox rates were measured by the labelled NH_4_^+^ with ^15^N [24]. The substrates (^15^NH_4_^+^ (^15^N at 99.0%) and ^14^NO_2_^−^) were added at a molar ratio of 1:1, and the final concentration was 40 mg·N/L. Mineral medium compositions were kept the same for all the influent water of different bioreactors.

Before adding the substrate, 48 h of pre-culture was performed without any composition to remove the original N. The argon gas aeration was performed incessantly to remove oxygen. Experiment with sole ^15^NH_4_^+^ substrate was used as controls in order to exclude the effect of DNRA, and the microbial activities at the end of the experiment were stopped by an addition of ZnCl_2_ (7 mol/L). The labelled samples were transported on ice to the Third Institute of Oceanography, Ministry of Natural Resources (Xiamen, China) for measuring N_2_. In this incubation experiment, denitrification and anammox were the two pathways to generate N_2_. The denitrification process only uses ^14^NO_2_^−^ to produce ^28^N_2_ (^14^NO_2_^−^ → ^28^N_2_), while the anammox uses ^15^NH_4_^+^ and ^14^NO_2_^−^ to generate ^29^N_2_ (^15^NH_4_^+^ + ^14^NO_2_^−^ → ^29^N_2_). The production of ^29^N_2_ through coupled nitrification and denitrification could be subtracted by the controls. Additionally, the activities of four key N-related enzymes (Nar: NO_3_^−^ → NO_2_^−^, Hzs: NH_4_^+^ → N_2_H_4,_ Nir: NO_2_^−^ → NO, Amo: NH_4_^+^ → NH_2_OH) were measured using a ELISA kit (Jiangsu yilaisa Biotechnology Co., Ltd) according to manufacturer’s protocols.

### DNA extraction and quantitative PCR assay of denitrifying and anammox bacteria

To monitor the biotic and abiotic characteristics across the anammox enrichment, we collected samples from the four bioreactors every month. Thus, we have four replicates for statistical analysis. All the samples collected for molecular analysis were immediately stored at − 80 °C until DNA extraction. The DNA was extracted using a Power Soil DNA Isolation Kit (Mo Bio Laboratories, Carlsbad, CA, USA) with a grinding extraction method [31]. Only the extracted DNA determined by Nanodrop (NanoDrop One, Thermo Scientific, USA) with A_260/280_ around 1.8 and A_260/230_ above 2.0 were kept for the subsequent experiments. The DNA concentration was finally quantified using the fluorescent method (Qubit 4 Fluorometer, Thermo Scientific, USA). The abundance of denitrifying and anammox bacteria were quantified by the real-time quantitative polymerase chain reaction (qPCR) [32] analysis of the *nirS/K* and *hzsB* genes, respectively. The standard curve was prepared using plasmid DNA. Briefly, the extracted DNA was specificity amplified, cloned, and extracted the plasmid DNA. After further purification and quantification, the plasmid DNA was then serially diluted to construct a standard curve using SYBR Green method. In the same way as plasmid DNA amplification, the samples were quantified according to the constructed standard curve. All amplifications were performed in a 20-μL of reaction ecosystem, which included 10 μL of SYBR Green mix, 0.25 μL of primer (5 mM), 2 μL of template DNA (10 ng), and 7.75 μL of sterile deionized water. Final quantification only using qPCRs with slopes between 3.39 and 3.92, amplification efficiencies greater than 95%, and with a single peak on the dissolution curve.

### Amplicons sequencing and data analysis

We detected a clear enrichment of anammox bacteria after 150 days, and the monitored *hzsB* gene showed the highest copies at 371 days. Thus, we only used the samples collected at 150 and 371 days for subsequent experiments, and lake sediments before enrichment (i.e., 0 day) were also analyzed. The *nirS/K* and *hzsB* genes were used as molecular markers to investigate denitrifying and anammox bacteria, respectively [33, 34]. *nirK* or *nirS*-type denitrifiers only contain either *nirK* or *nirS* functional genes, but not both genes [35–37]. Previous studies have demonstrated that *hzsB* is present in all known anammox bacteria and exhibits high sequence conservation across different species and genera [19, 38]. We also investigated the overall bacterial community based on the sequenced V3-V5 regions of 16S rRNA gene. PCR amplifications were conducted in a 10-μL reaction volume with 10 U of Phusion High-Fidelity DNA Polymerase (NEB, Inc., USA) and 0.2 mM of each primer, 10 ng of DNA template. A summary of primer information and reaction conditions is presented in Table S3. The TruSeq® DNA PCR-Free Sample Preparation Kit (Illumina, USA) was used to determine the concentration of DNA PCR products, then combined equally and mixed fully. The constructed libraries of different genes were then sequenced on an Illumina HiSeq PE250 platform (Illumina, Inc., CA, USA) in Majorbio Bio-pharm Technology Co., Ltd. (Shanghai, China). Sequencing data were analyzed according to our previously study [29]. Briefly, Trimmomatic v0.33 was used for quality control [39], and sequences without primer fragments were removed using FASTX_Toolkit, any sequence containing an ambiguous base ('N') was also removed. UCHIME was used to identify and remove chimeric sequences. Framebot software was used to correct frameshifts caused by sequence errors when creating OTUs on a Linux system using Uparse cluster.

### Metagenome sequencing and data analysis

To give a more comprehensive profile of N cycling genes before and after the anammox enrichment, 6 samples from two time points (0 and 371 days, three replicates for each time) were selected for shotgun metagenome sequencing. DNA fragment libraries were constructed using 1 μg of high-quality DNA by NEXTFLEX Rapid DNA-Seq Kit (Bioo Scientific, USA), and then sequenced using a Novaseq6000 platform at the Shanghai Majorbio Bio-pharm Biotechnology Co., Ltd. (Shanghai, China). The raw data was filtered using Trimmomatic v0.38 to remove reads containing more than three ambiguous nucleotides with an average quality score < 20, and artificial duplicate reads were also removed [39]. Then, the high-quality raw reads were optimized by trimmed and assembled into contigs using MEGAHIT (v1.2.9). Only the contigs ≥ 500 bp were kept for subsequent analysis. The open reading frames (ORFs) of all the assembled contigs were predicted using MetaGene, and translated into amino acid sequences. Then, the putative protein-coding sequences were searched against the KEGG database and eggNOG database using DIAMOND blastx. Trans Per Million (TPM) values were used to determine whether the difference before and after enrichment was significant. The Metabat2 (v2.12.1) and MaxBin2 (v2.2.5) were used to binned each contigs from each sample. The original bins were consolidated and improved with Bin_refinement and Reassemble_bins module in metaWRAP. CheckM (v1.0.12) was used to estimate the completeness and contamination of the metagenome-assembled genomes (MAGs), and only the MAGs with completeness > 50% and contamination < 10% were kept for the subsequent analyses. To perform taxonomic annotation, the ORFs of each gene were extracted and searched against the NCBI-NR database and KEGG database using DIAMOND blastx with an *e* value ≤ 10^−5^. The genes annotated by KEGG were further assigned to KO, pathway, and module, respectively.

### Statistical analysis

The principal coordinates analysis (PCoA) was used to visualize the differences between denitrifying and anammox bacterial communities based on the Bray–Curtis distances [40]. The β-nearest taxon index (βNTI), which calculated by the picante R package [41], was calculated to infer the community assembly processes. A heatmap was constructed by the pheatmap R package to show the normalized abundances of OTUs, and the average abundances and evolutionary distances of OTUs were used for column and row clustering, respectively. A Venn diagram was constructed using the R package euler and plot to compare composition of microbial OTUs among stages. We also conducted Linear discriminant analysis Effect Size (LEfSe) (http://huttenhower.sph.harvard.edu/galaxy) to identify discriminative taxonomic differences among stages. A phylogenetic Molecular Ecological Network Analysis (MENAP) pipeline (http://ieg4.rccc.ou.edu/mena) was used to construct co-occurrence correlation networks based on Random Matrix Theory (RMT) methods. Only the OTUs connected with denitrification and anammox were retained for subsequent calculations [42]. We assessed the stability of microbial communities by calculating the robustness and vulnerability. The correlation between dominant taxa and environmental factors were measured using the Spearman coefficient. Mantel tests were conducted to find which physicochemical factors significantly correlate with bacterial communities [29]. Using R package plspm, partial least squares path modeling (PLS-PM) was performed to determine the contribution of denitrifying and anammox bacteria to N removal. We used the R randomForest package to identify keystone taxa using a classification random forest analysis. The relative abundance of each functional gene category (KEGG functional gene annotated level 2) was also calculated for random forest analysis [42].

## Results

### Stage-dependent N removal across the anammox enrichment with lake sediments

**Fig. 1:**
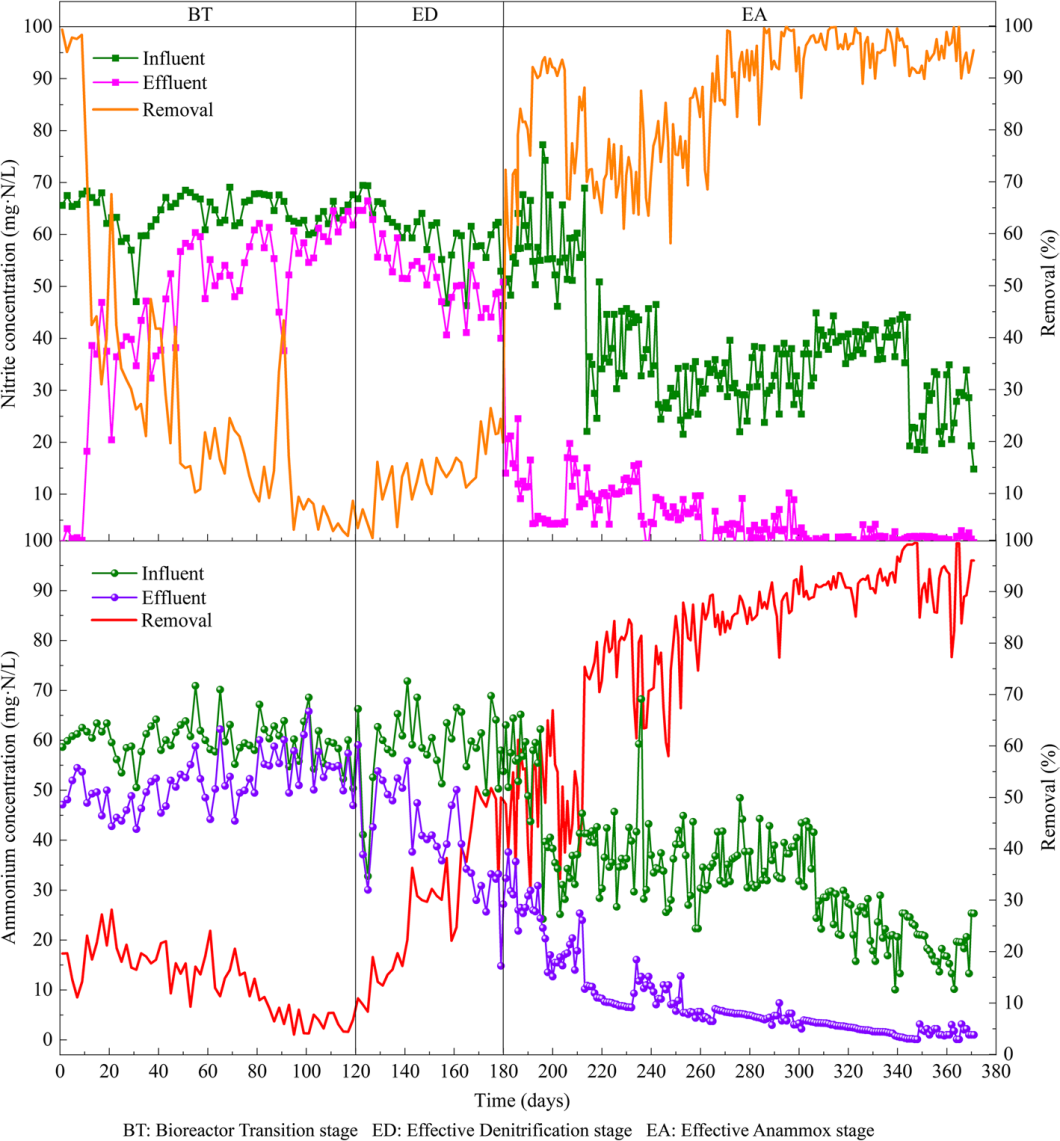
Nitrogen conversion and removal efficiency across the start-up of anammox enrichment with lake sediments in bioreactor systems

The start-up of anammox enrichment with lake sediments in bioreactor systems could be divided into three stages (i.e., 1–120 days, 121–180 days, and 181–371 days) according to the continuously monitored N removal efficiency (Fig. 1). The first stage showed a relatively low NH_4_^+^ and NO_2_^−^ removal efficiencies. This stage was mainly for the bacteria inoculated from lake sediments to adapt to the conditions given in the bioreactors, and thus called bioreactor transition (BT) stage. During this stage, the HRT was 48 h and the concentrations of NH_4_^+^ and NO_2_^−^ were 60 mg·N/L with a molar ratio of 1:1. The second stage was characterized by obvious denitrification and ammonia oxidation, the removal efficiencies of NH_4_^+^ and NO_2_^−^ increased to 48.0% and 27.5%, respectively. This stage was thus called effective denitrification (ED) stage, and the minimum effluent concentrations of NH_4_^+^ and NO_2_^−^ decreased to 25.7 mg·N/L and 42.3 mg·N/L, respectively. As appropriate substrate concentration could promote the enrichment of anammox, the HRT at this stage was gradually decreased from 48 to 24 h to ensure a stable substrate. The third stage was characterized by a clear increase of anammox activity when the concentrations of NH_4_^+^ and NO_2_^−^ (a molar ratio of 1:1) decreased from 60 to 35 mg·N/L, and thus called effective anammox (EA) stage. The HRT at this stage was kept the same as the second stage (i.e., 24 h). The effluent concentrations of NH_4_^+^ and NO_2_^−^ were significantly decreased at day 260, and the maximum removal efficiencies reached up to 85.9% and 95.3%, respectively. At the BT and ED stage, the products of NO_3_^−^ could be observed in the effluent with an average concentration of 21.8 mg·N/L (Figure S2). The NO_3_^−^ product at EA stage decreased from 42.3 mg·N/L to 8.3 mg·N/L, and then kept at a stable concentration around 7.0 mg·N/L during rest days.

### Quantification of anammox and denitrifying bacteria and their N removal rates

**Fig. 2:**
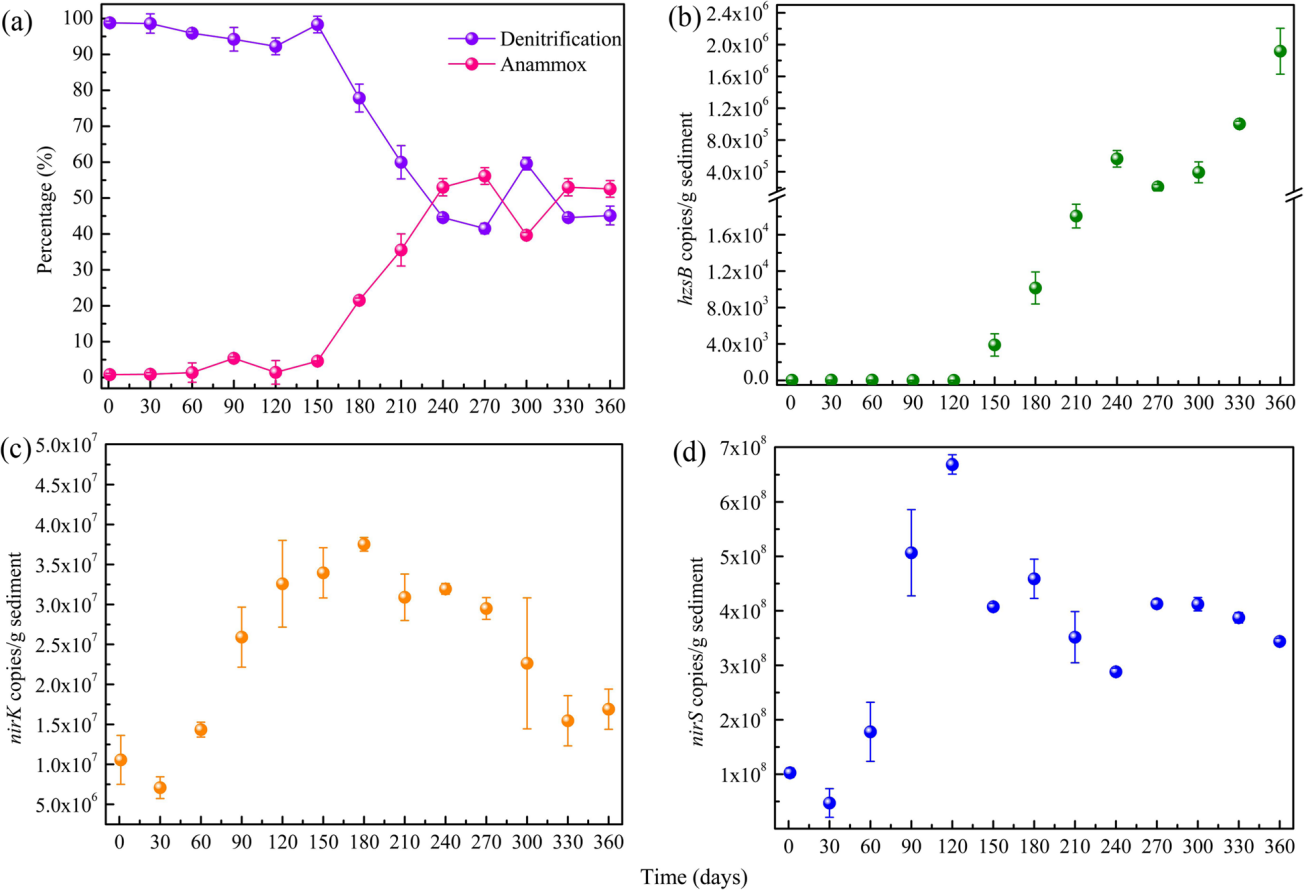
Quantification of the anammox and denitrification across the anammox enrichment with lake sediments in bioreactor systems. Potential denitrification and anammox ratio determined by the ^15^NH_4_.^+^ labelling analysis (**a**). Copy numbers of the *nirS/K* and *hzsB* genes determined by real-time PCR (**b**, **c**, **d**)

The quantity of anammox and denitrifying bacteria in the bioreactors was evaluated by the qPCR. The abundances of anammox bacteria (*hzsB*), *nirK*, and *nirS*-type denitrifiers in the collected lake sediments were 34.57, 7.09 × 10^6^ and 4.72 × 10^7^ copies/g dry sediments, respectively (Fig. 2). In the bioreactor enrichment, anammox bacteria kept at a relatively low abundance (≤ 3.9 × 10^3^ copies/g dry sediment) throughout the first 120 days (Fig. 2b). The abundances of denitrifying bacteria (*nirS/K*) were initially increased, but decreased as the operation time (Fig. 2c, d). However, the *nirS* gene copies were always greater than the *nirK* gene copies during the enrichment. Correspondingly, the ^28^N_2_ produced by denitrification kept at a relatively high level (> 90%) before 120 days (Fig. 2a). From days 120 to 360, the quantity of *nirS/K* genes copies began to decrease, while the *hzsB* gene copies increased considerably and the anammox activity kept a relatively high level. The maximum value of anammox bacteria can reach to 1.92 × 10^6^ copies/g dry sediment. Isotopic analysis showed that the ^29^N_2_ produced by anammox bacteria increased to 56.14% (Fig. 2a), indicating an enhanced N removal by anammox process. In contrast, the ^28^N_2_ produced by denitrification decreased significantly from 77.85% to 44.58% from 180 to 360 days.

### Anammox and denitrifying bacterial diversity and composition

The denitrifying and anammox bacterial communities collected at the 0, 150, and 371 days were analyzed by the *nirS/K* and *hzsB* genes amplicons sequencing. The Shannon and Chao1 of anammox and *nirS*-type denitrifying bacteria were significantly (*p* < 0.05) higher at the stages of BT than that of ED and EA stages. However, *nirK*-type denitrifying bacteria showed significant (*p* < 0.05) lower diversity at the BT stage than that of ED and EA stages (Fig. S3a). PCoA revealed significant (*p* < 0.05) differences in anammox and denitrifying bacterial communities among different stages (Figure S3b). The quantification of community assembly processes indicated that the deterministic variable selection (βNTI > 2) was the dominant process governing the community assembly at the ED and EA stages, whereas the stochastic process (|βNTI|< 2) played a vital role at the BT stage (Figure S3c).

**Fig. 3:**
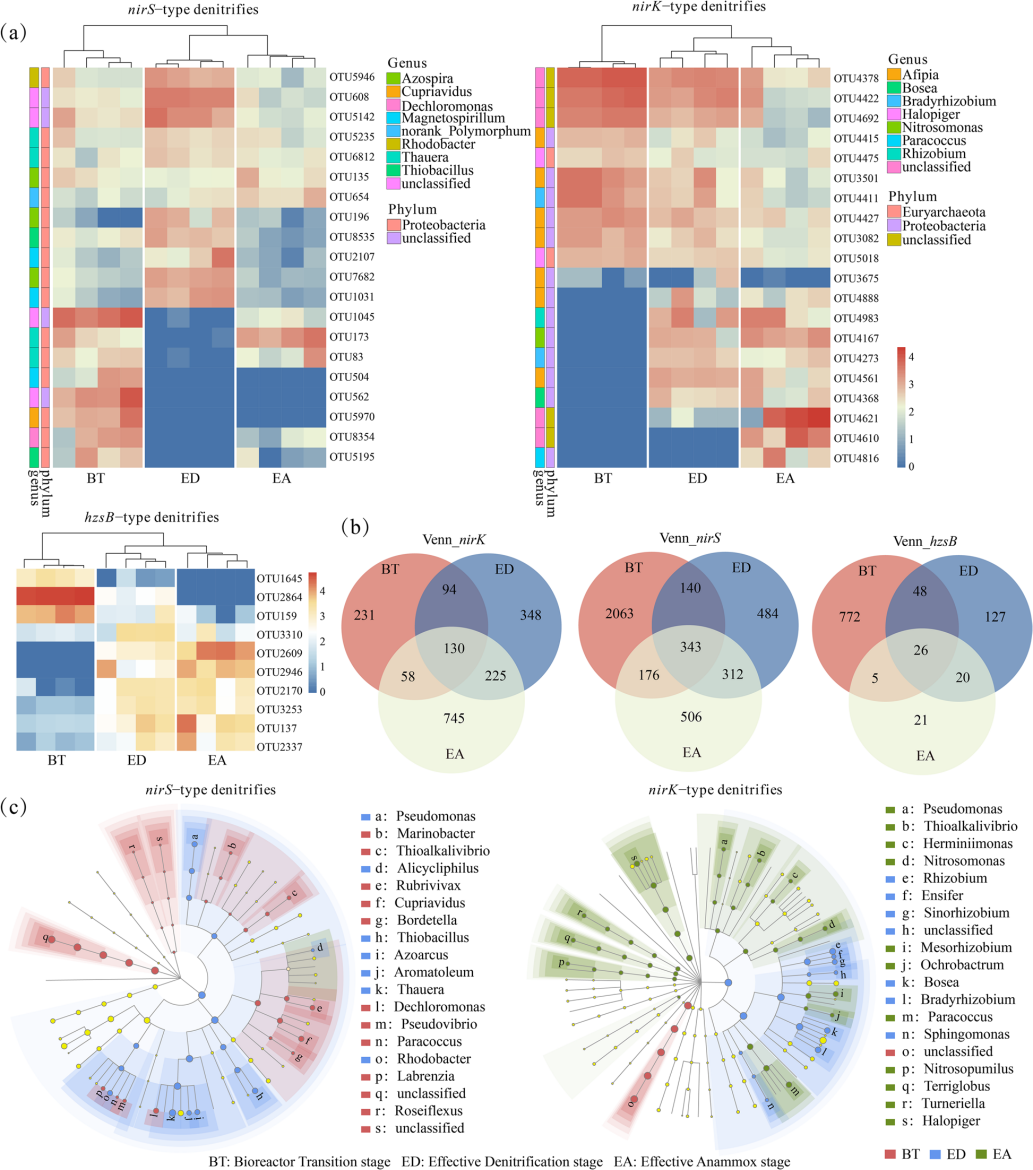
Composition of denitrifying and anammox communities as depicted by amplicon (*nirS/K* and *hzsB* genes) sequencing across the anammox enrichment with lake sediments in bioreactor systems. Heatmap showing the relative abundance of the top 20 OTUs of *nirS/K*-type denitrifiers (**a**). Venn diagram showing the unique and shared OTUs among different stages (**b**). Taxonomic cladogram showing the discriminative taxa among different stages based on the linear discriminant analysis (LDA-score > 2.0) with effect size measurements (LEfSe) (**c**)

During the ED and EA stages, the enriched *nirK*-type denitrifiers including *Afipia*, *Halopiger*,* Rhizobium*,* Bradyrhizobium*, and *Mesorhizobium* (Figure S4). The *nirS*-type denitrifying bacteria were dominated by *Thauera*,* Azospira*,* Rhodobacter*,* Thiobacillus*, and *Azoarcus* during the ED stage (Figure S4). However, the denitrifying community shifted considerably during this enrichment. The top 20 OTUs showed that the *nirS/K*-type denitrifying bacteria were considerably different among the three stages (Fig. 3a). Interestingly, the *nirS*-type OTU 173 and OTU 83 (*Thauera*) were initially decreased, but increased as the operation time, while the *nirK*-type OTU 4621 and OTU 4610 (unclassified), OTU 4888 and OTU 4561 (*Afipia*), and OTU 4816 (*Paracoccus*) were considerably enriched at the EA stage. The LEfSe analysis indicated significantly different genera between BT and ED stages (Fig. 3b Table S4). For example, *Thauera, Azoarcus* (LDA score > 4.0, *p* < 0.01), *Pseudomonas*,* Thiobacillus*, and *Rhodobacter* (LDA score > 4.0, *p* < 0.05) were significantly enriched at the ED stage (Fig. 3b Table S4). Overall, there were 18 bacterial taxa showing distinct abundances among the three stages (LDA score > 4.0, *p* < 0.05) (Table S4). The denitrifying bacteria such as *Halopiger* and *Mesorhizobium* significantly enriched at the EA stage, whereas *Rhizobium* and *Bradyrhizobium* significantly enriched at the ED stage (Fig. 3b). The abundance of *Candidatu*s Jettenia increased along the operation time (Figure S4). However, 50–90% of anammox bacteria detected were unclassified at the genus level. The phylogenetic analysis of 16S rRNA gene indicated that the relative abundances of anammox and denitrifying bacteria changed with the reactor operation at the phylum level (Figure S5). Specifically, the relative abundances of Chloroflexi, Acidobacteria, Planctomycetes and Gemmatimonadetes increased.

**Fig. 4:**
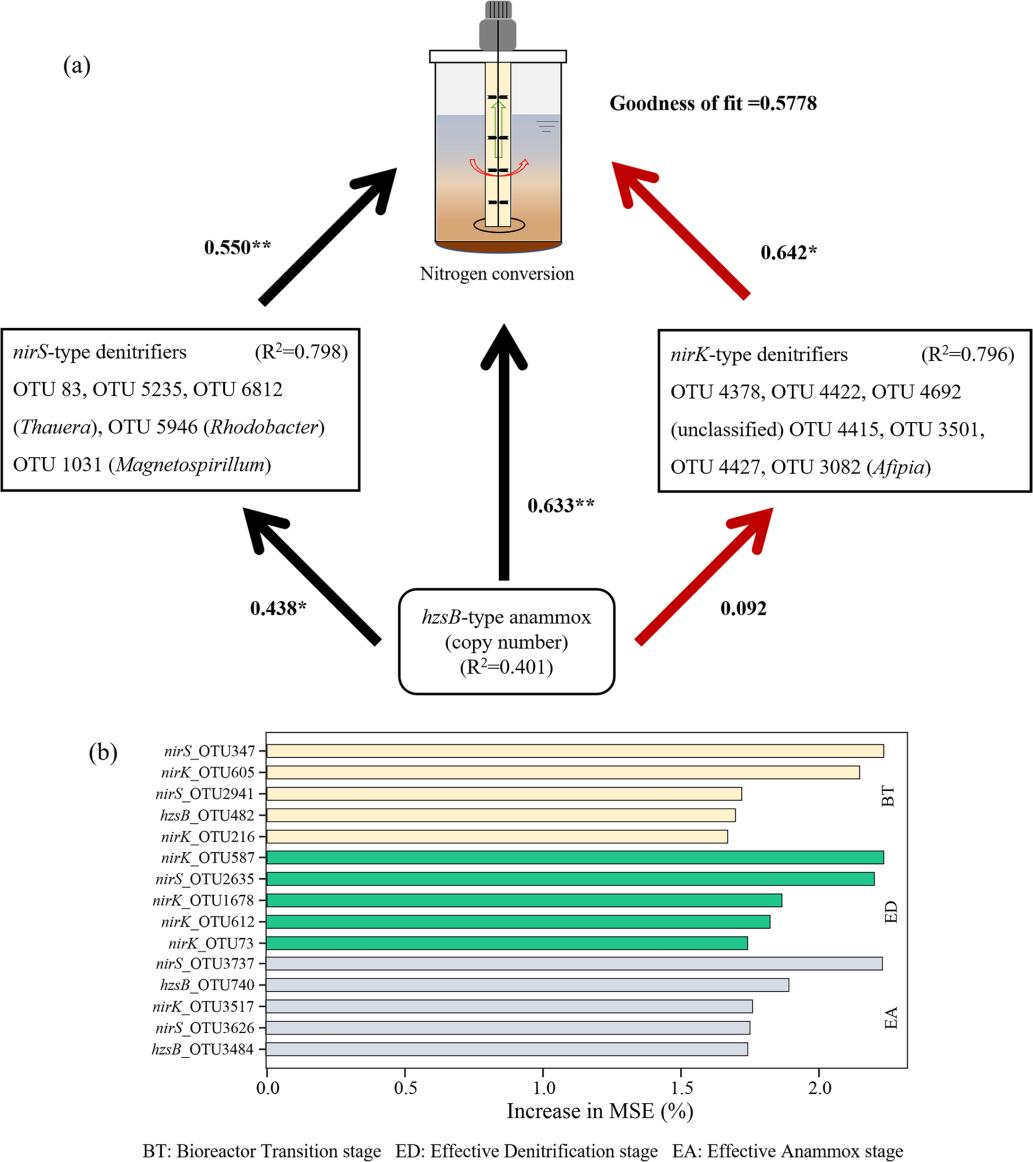
Cascading relationships of the denitrification and anammox functions. Path diagrams estimating the effects of *nirS/K*-type OTUs on the anammox enrichment with lake sediments in bioreactor systems (**a**). Black and red lines represent positive and negative effects, respectively. Numbers adjacent to the arrows are standardized path coefficients. The arrows refer to unidirectional causal relationships (*p < 0.05; **p < 0.01). The keystone taxa identified to be important for predicting the denitrification and anammox functions at different stages (**b**)

The Venn diagram showed that *nirK*-type denitrifiers had more unique OTUs than *nirS*-type denitrifiers at the EA stage (Fig. 3c), and the number of unique anammox OTUs decreased across the enrichment. The PLS-PM indicated that the *nirS* and *nirK*-type OTUs contributed positively (path coefficients = 0.549, *p* < 0.01) and negatively (path coefficients = 0.642, *p* < 0.05) to the anammox bacteria, respectively (Fig. 4a). Moreover, *Thauera*,* Rhodobacter*,* Magnetospirillum* and *Afipia* could be the key genera affecting the coupling of anammox and denitrification (Fig. 4a, Figure S6).

The random forest analysis indicated that the *nirS*-type keystone taxa contributed greatly to N removal function prediction at the BT stage (e.g., OTU347) and EA stage (e.g., OTU3737), which accounting for 2.23% and 2.15%, respectively (Fig. 4b). Thus, the *nirS*-type keystone taxa played important roles for the enrichment of anammox bacteria, whereas the anammox keystone taxa were more important at the EA stage than other stages (Fig. 4b).

### Interactions and community stability of denitrifying and anammox bacteria

**Fig. 5:**
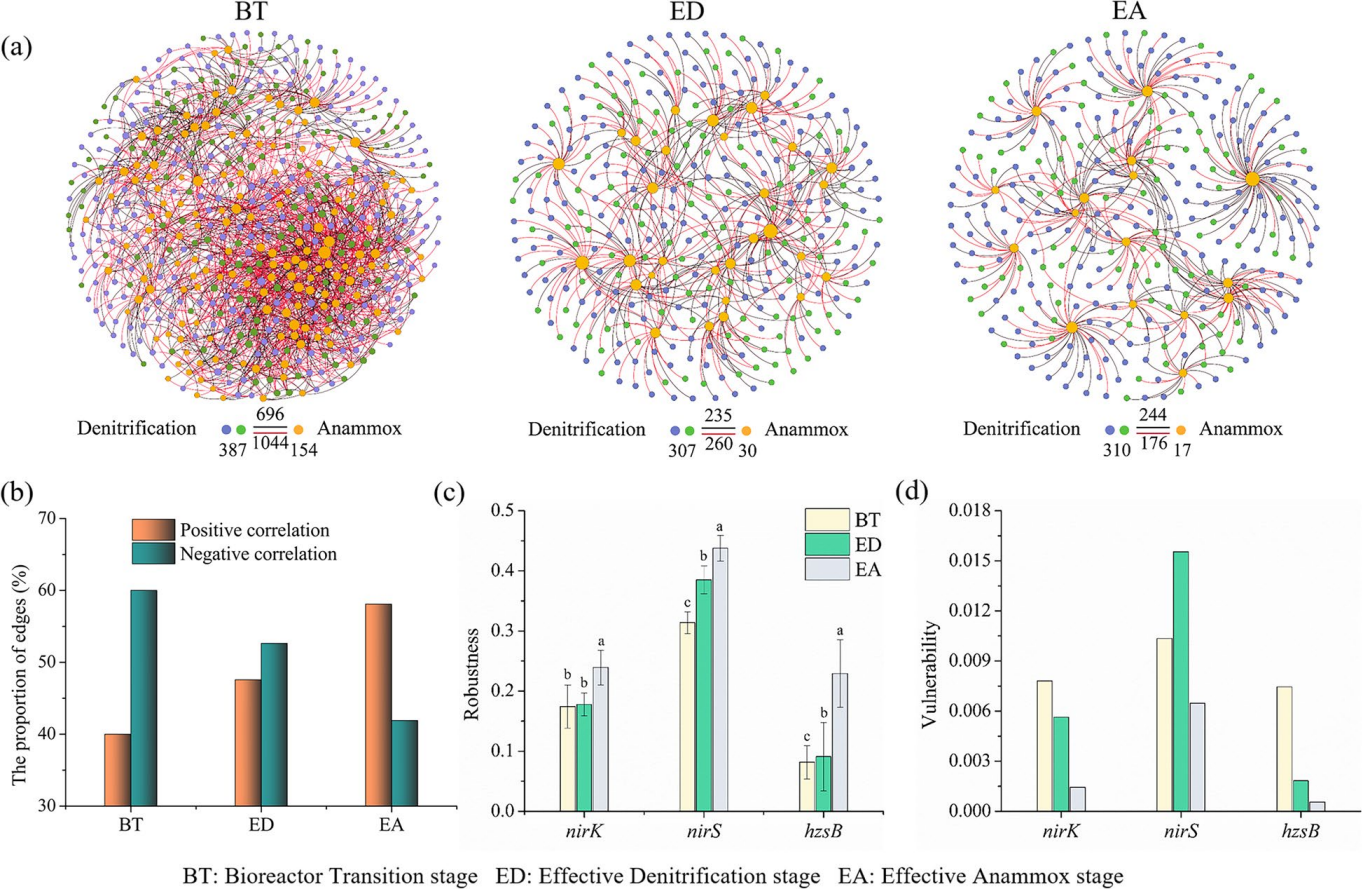
Co-occurrence networks of denitrifying and anammox bacteria and their stability comparison among different stages across the anammox enrichment with lake sediments in bioreactor systems. Co-occurrence networks of denitrifying and anammox bacteria constructed based on the sequenced functional genes of *nirS/K* and *hzsB* (**a**). The node size represents the degree of each OTU. The black and red links represent positive and negative correlations, respectively. A summary of node-link statistics was also given, and numbers represent the nodes or links. The positive and negative proportion of the network correlations (**b**). Community stability as depicted by the robustness (**c**) and vulnerability (**d**), respectively. Robustness is measured as the proportion of the remaining species in community after 50% of the nodes were randomly removed, and vulnerability is measured as the maximum vulnerability of nodes in each network

We constructed denitrifying and anammox networks to explore interspecies interactions (Fig. 5a). The results showed that the number of nodes and links for denitrifiers were much more than that of anammox bacteria, indicating much more connected and complicated denitrifying community networks. The BT stage showed the highest number of nodes and links, and > 60% connections were negative, indicating a clear competition between denitrifiers and anammox bacteria at this stage (Fig. 5b). However, the positive connections increased from BT to EA stages, and the highest ratio of positive connections at the EA stage indicated a cooperative relationship formed between anammox and denitrifying bacteria.

We further calculated robustness and vulnerability to evaluate the community stability. The results showed that denitrifiers and anammox bacteria showed significantly higher robustness at the EA stage than at the BT and ED stages (Fig. 5c), whereas the vulnerability decreased considerably across the anammox enrichment except the *nirS*-type denitrifiers (Fig. 5d). Thus, the stability of denitrifying and anammox communities increased gradually across the anammox enrichment.

### The coupling between anammox and denitrification

**Fig. 6:**
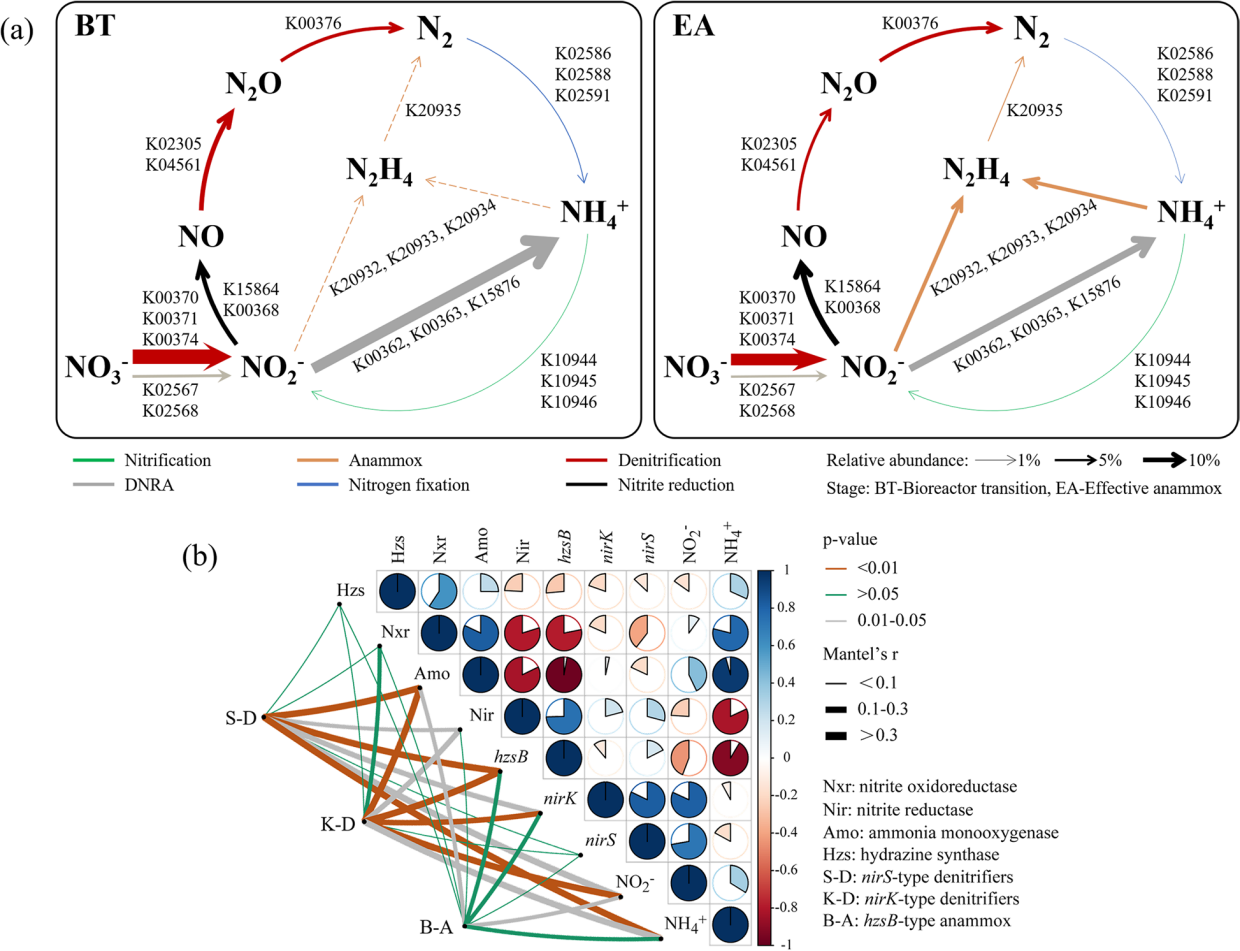
Nitrogen cycling genes’ abundance, functional categories and bacterial correlations with environments. The relative abundance of the nitrogen cycling genes before and after enrichment as depicted by metagenome sequencing (**a**). Genes abundances were normalized into transcripts per million (TPM) counts. The dashed line indicated the undetected pathway. Pairwise Spearman’s correlation matrix summarized the relationships between bacteria and environmental factors (**b**). Correlations were shown as pie charts, which were determined by the Mantel tests

The metagenome sequencing indicated that the anammox related genes showed the highest abundance after the start-up enrichment (i.e., EA stage, Fig. 6a). Specifically, the genes in responsible for NO_2_^−^ reduction (NO_2_^−^ → NO) showed much higher abundance at the EA stage than that before enrichment, but the genes for other denitrification processes (e.g., NO_3_^−^ → NO_2_^−^, NO → N_2_) were lower in the EA stage than that before enrichment. The relative abundance of denitrifying and anammox genes showed similar high abundance after the start-up of enrichment in the bioreactor system, but the denitrifying genes absolutely overwhelmed the anammox genes (undetected) before enrichment (Fig. 6a). Similarly, the N metabolic pathway predicted from the 16S rRNA gene sequences also indicated that the denitrifying genes were initially increased, and then decreased across the enrichment (Figure S7).

The partial Mantel tests were used to explore the relationships between N transformation and the related bacterial groups. The composition of *nirS*-type denitrifiers was significantly affected by NH_4_^+^ (*r* > 0.3, *p* < 0.01), whereas *nirK*-type denitrifiers were mainly affected by the NO_2_^−^ (*r* > 0.3, *p* < 0.01) (Fig. 6b). Moreover, the *hzsB* gene was significantly correlated with both *nirS*-type (*r* > 0.3, *p* < 0.01) and *nirK*-type (*r* > 0.3, *p* < 0.01) denitrifying bacteria. Additionally, the anammox communities, which were significantly (*p* < 0.05) correlated with the concentration of NO_2_^−^, were more likely regulated by *nirK*-type denitrifiers. Thus, the denitrifying bacteria could be a primary factor that affecting the abundance of anammox bacteria.

### Metabolism potential changed before and after the anammox enrichment

**Fig. 7:**
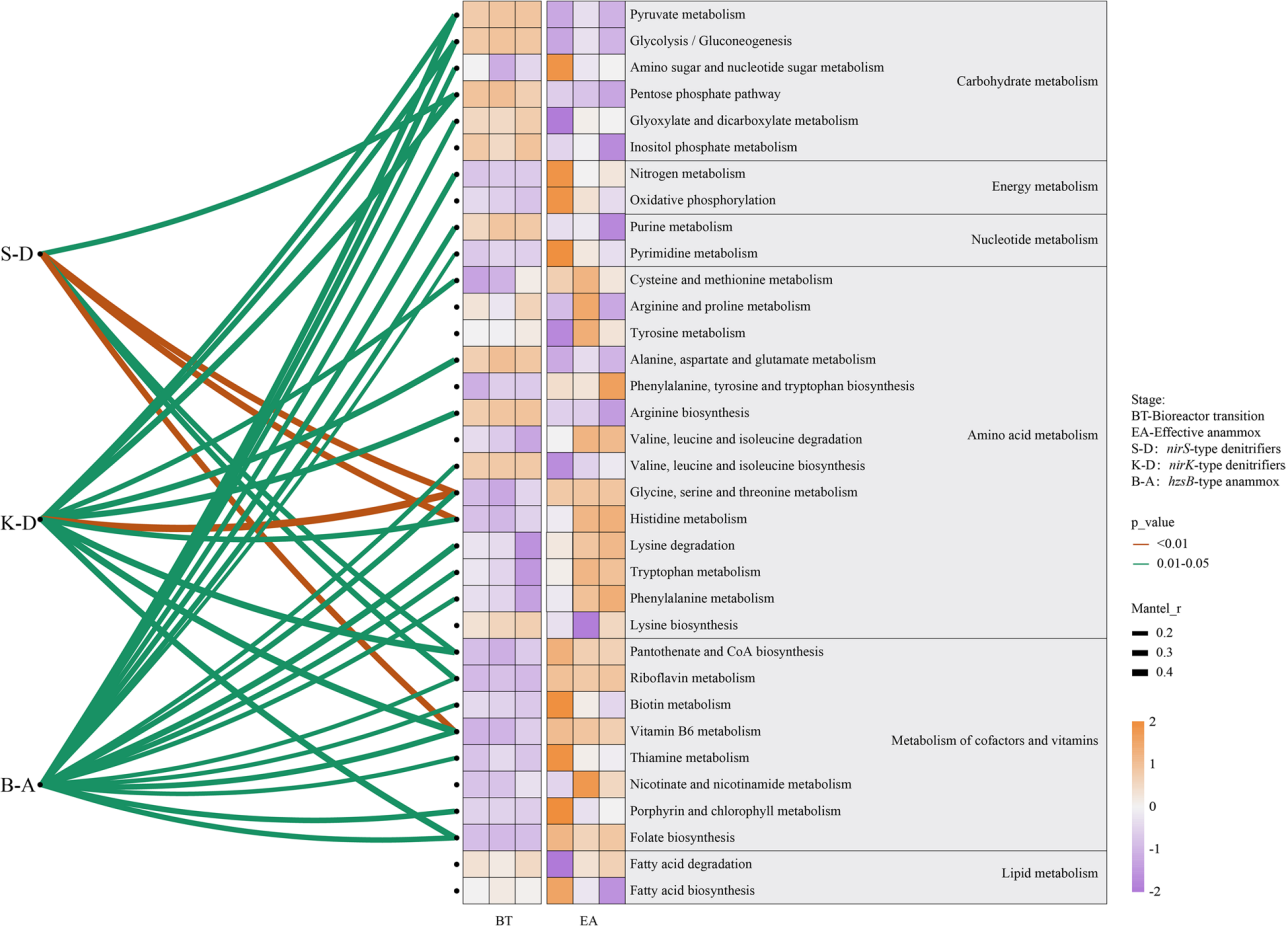
Metabolism pathways of KEGG modules before and after anammox enrichment. The abundance of KEGG modules were calculated by homogenization and normalized into TPM counts. Pairwise Spearman’s correlation matrix summarized the relationships between bacteria and metabolism pathways, which were determined by the Mantel tests

Base on the metagenome sequencing, we further explored how the denitrifying bacteria responded to nutrient starvation by examining the KEGG metabolism pathways. We found denitrification could account for 40% of the total amount of bacteria (Figure S8). The abundance of energy metabolism increased considerable at the EA stage compared with that before enrichment, which was in line with the nutrient starvation state at this stage. The metabolic related to nucleotides, amino acids, cofactors, and vitamins were also much higher than those before enrichment, whereas the carbohydrates metabolism was decreased after the enrichment (Fig. 7). To determine how individual metabolisms contributed to the observed denitrifying and anammox bacteria, KEGG modules were employed to explore the metabolism patterns before and after enrichment. When denitrifying bacteria subjected to starvation stress at the EA stage, a higher abundance of nucleotide sugar metabolism and amino sugar oxidation were observed. Additionally, the abundance of oxidative phosphorylation, which in responsible for ATP biosynthesis to maintain basic cellular reactions, was much higher at the EA stage than that before enrichment. A relatively high abundance of pyrimidine metabolism was also observed at the EA stage. The anammox bacteria enriched (EA stage) showed higher abundance of metabolism and biosynthesis of the amino acids. The amino acids metabolism pathways for degradation and synthesis of amino acids were always highly expressed at the stage EA after the anammox enrichment. The metabolic functions and microbial interactions predicted based on the 16S rRNA gene indicated that the signaling molecules and interactions accounted for the highest proportion of the metabolism category (Figure S9). Anammox communities showed significant correlations (*p* < 0.05) with numerous amino acid, cofactors, and vitamins metabolisms. However, denitrifying communities showed significant relationship with glycine, serine, histidine, and threonine metabolisms (Fig. 7). The correlation analysis based on the Pearson was further conducted to reveal the relationships between amino acids, cofactors, vitamins and denitrifying, anammox related genes (Figure S10). Our results showed that anammox related genes (*hzsA/B/C*) and *hao* gene were positively correlated with the majority of amino acids, cofactors, vitamins. Among these, valine, leucine and isoleucine biosynthesis, glycine, serine and threonine metabolism, histidine metabolism, and folate biosynthesis had significant (< 0.01) effect on *hzsA/B/C* and *hao* gene (Figure S10).

**Fig. 8:**
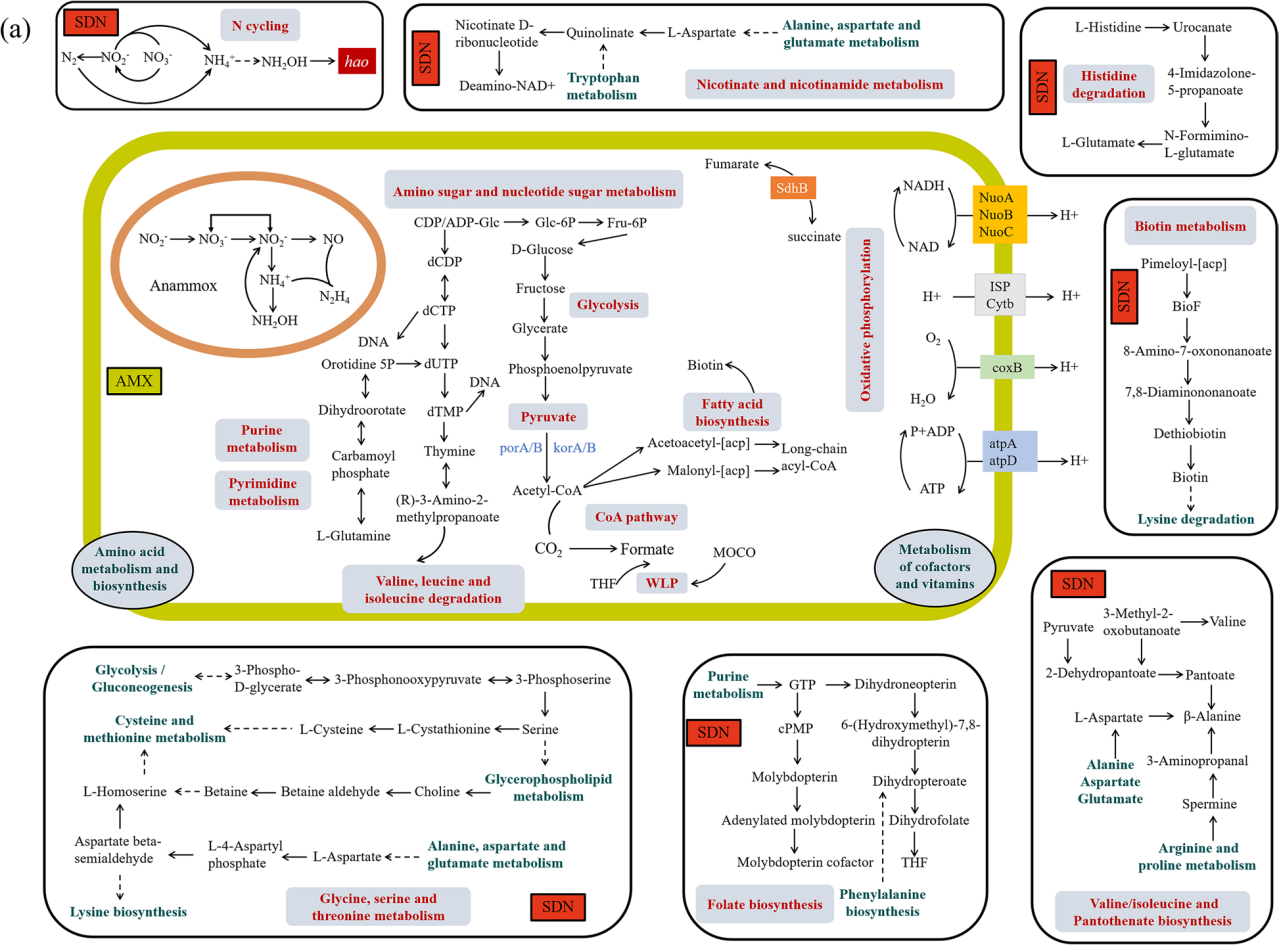
Potential metabolisms and nitrogen cycling pathways coupling between Candidatus *Jettenia* (AMX) and symbiotic denitrifiers (SDN). Genome statistics information are listed in Table S5

Contigs were subsequently binned into MAGs, and 20 MAGs were recovered from the three samples at the EA stage. However, only one MAG was classified as *Candidatus* Jettenia (AMX) (Table S5). To explore the metabolic pathways after the enrichment, the symbiotic denitrifiers (SDN) MAGs were selected for further analysis (Table S5). The genome-based ecological model indicated that AMX encoded nearly complete pyruvate metabolism, glycolysis, CoA pathway, amino sugar, and nucleotide sugar metabolism (Fig. 8). However, the AMX genome lacked many cofactors and vitamins required for oxidative phosphorylation, CoA pathway, CO_2_ fixation, and fatty acid biosynthesis (Figure S12a). Furthermore, AMX were missing pathways for the synthesis of numerous crucial amino acids (Figure S12a), indicating AMX populations need acquire essential nutrients from other microorganisms for their growth in the anammox enrichment system. SDN harbored complete pathways for the biosynthesis of most amino acids, especially some with high biosynthetic cost, including the production of valine, serine, threonine, betaine, and cysteine (Figure S12a). SDN encoded the biosynthesis pathways for most AMX purine and pyrimidine metabolism precursors, including glutamine biosynthesis and valine degradation. SDN encoded all related genes for the tetrahydrofolate (THF) synthesis, molybdenum cofactor biosynthesis (MOCO), and biotin biosynthesis, which could cross feed with AMX. Similarly, SDN also harbored genes to facilitate NAD production. To identify genes functions that were enriched in the above MAGs, we compared the functional difference of each MAG at the level of COG class. SDN were enriched in COG class compared with AMX, including coenzyme transport and metabolism, and amino acid transport and metabolism (Figure S11). Additionally, SDN encoded the genes *narGHI* might function for the NO_2_^−^ loop (Figure S12b). NO_2_^−^ reduction NH_4_^+^ related genes (*nirB*, *nrfH/A*) had highly ubiquitous in symbiotic denitrifiers (SDN) after the enrichment (Figure S12b). Interestingly, the *hao* gene is ubiquitously detected in SDN and AMX, suggesting the *hao* may play a vital role in anammox metabolism under anaerobic conditions. The NO_2_^−^ could be reduced to NH_4_^+^, and the NH_4_^+^ could be oxidized to produce the *hao* gene, which indicated a “NH_4_^+^ loop”. Together, the NO_2_^−^ loop or NH_4_^+^ loop might affect the anammox activity.

## Discussion

Understanding the ecological interactions and underlying mechanism of anammox and denitrification in lake ecosystems will greatly enhance the N removal to control lake eutrophication. However, the application of microbial N removal in eutrophic lakes lags far away from that used in wastewater treatments. Although the anammox has been reported in freshwater lake for more than 15 years [3], our understanding of anammox in lakes is still mainly restricted to field investigations [43–47]. So far, we still lack a successful enrichment of anammox bacteria from lake environments [4, 45–47] to understand the anammox mechanism in lakes. A major difficulty for such enrichment is the denitrification in lake water–sediment interface always inhibits the start-up of anammox. Thus, establishing a quick start-up of anammox enrichment with lake sediments is of great importance for studying N removal in eutrophic lake ecosystems [48, 49]. This study successfully started up the anammox enrichment with eutrophic lake sediments, and the maximum removal of NH_4_^+^ and NO_2_^−^ reached to 85% and 95%, respectively. We attempted to identify additional roles of denitrifiers in anammox performance with respect to coupling within the anammox in terms of amino acid metabolism, cofactors, and vitamin biosynthetic. We found that the ecological interactions, especially through niche differentiation and cooperation between anammox and denitrification could promote the N removal in lake ecosystems. This study not only highlights a cross-feeding of the anammox and denitrifying bacteria, but also implies denitrifying potentials in anammox activity and growth.

According to the continuously monitored N removal efficiencies, the start-up of anammox enrichment with lake sediments also could be divided into three stages characterized as bioreactor transition (BT, 1–120 days), effective denitrification (ED, 121–180 days), and effective anammox (EA, 181–371 days) [50]. The N removal efficiency was significantly increased across the three stages, indicating the microbial N removal in natural lake environments is limited. The anammox enrichment conditions given in the bioreactors resulted in disappearance of most aerobic microorganisms derived from lake sediments, and heterotrophic nitrifying bacteria (HNB) (e.g., *Pseudomonas, Bosea*) tended to consume NH_4_^+^ through the inorganic oxidation [51]. Some HNB also could consume NO_2_^−^ through the complete denitrification process. Thus, the N removal efficiency was only about 20% at the first stage (BT) and the anammox activity was relatively low. Then, the quantity of *nirK/S* and *hzsB* genes copies showed a slow increase at the second stage (ED), suggesting an initial coupling between denitrification and anammox. The NO_2_^−^ reduction process including three pathways, i.e., NO_2_^−^ → N_2_ (denitrification), NO_2_^−^ → NH_4_^+^ (DNRA), NO_2_^−^$\leftrightarrow$ NO_3_^−^. The genes regulating the process of NO_2_^−^$\leftrightarrow$ NO_3_^−^ showed relatively higher abundance during the enrichment (all the three stages). The *nirS/K* genes are partially involved in regulating both anammox and denitrification [52]. *nirK* gene is highly sensitive to substrate concentration. Previous studies found that NO_2_^−^ generated in periplasmic space was not metabolized in time and excreted out of the cell, which may be related to the high expression of *nirK* gene [53]. Generally, bacteria with *nirK* gene have the ability of partial denitrification, but such *nirK*-type bacteria can also result in nitrite accumulation [54]. NH_4_^+^ could be oxidized by the *hao* gene. The *hao* gene was supposed to regulate the process of NO_2_^−^ → NO in anammox metabolism. At the BT and ED stage, the product ratio of NO_3_^−^-N: NH_4_^+^-N was larger than 0.26, confirming that beside anammox there also have some other processes could generate NO_3_^−^ (i.e., NO_2_^−^$\leftrightarrow$ NO_3_^−^). The ED stage is generally characterized by an obvious denitrification, ammonia oxidation, as well as anammox. At the third stage (EA), the anammox bacterial abundance and its N removal efficiency were all increased significantly, indicating a successful start-up of anammox enrichment. Moreover, the removal ratio of NO_2_^−^-N: NH_4_^+^-N was larger than 1.32, confirming that NO_2_^−^ could be removed through the processes of denitrification and anammox [43].

Chloroflexi, Acidobacteria, Planctomycetes and Proteobacteria were the dominant groups after enrichment. Actually, Acidobacteria could use multiple electron acceptors to reduce Fe or single carbon compounds [55], Proteobacteria might perform partial nitrification result in NO_2_^−^ accumulation [56], and Chloroflexi was previously identified as the dominant bacteria in anammox and denitrification reactors. The relatively simple anammox community diversity as indicated by the *hzsB* gene indicated species-specific niche differentiation and competition across the enrichment [4]. Meanwhile, the diversity of *nirS*-type denitrifying community declined, but that of *nirK*-type community increased significantly across the enrichment. As bacterial diversity tends to increase under environmental stress [20], suggesting the enrichment conditions given are beneficial for the anammox and *nirS*-type denitrifying bacteria. Moreover, the microbial community across enrichment is generally shifted from a complex to a simple and stable state [57]. Thus, *nirS*-type denitrifying bacteria enriched under the suitable conditions [20] also could help the enrichment of anammox bacteria. The dominant *nirS/K*-type denitrifying bacteria detected herein were mainly affiliated to Proteobacteria, which not involved in anammox but usually coexist with anammox Planctomycete [58]. We found that *Thauera* was the most important biotic group shaping the anammox community, which is consistent with previous studies [59, 60], as the *Thauera* really could enhance the anammox rate [61]. In addition, some other enriched denitrifying genera (e.g., *Rhodobacter, Magnetospirillum, Bradyrhizobium* and *Afipia*) were also generally in agree with previous studies, and such denitrifiers could relieve the inhibition of organic carbons and decrease the toxicity of NO_2_^−^ for anammox bacteria [62, 63]. Additionally, *Pseudomonas* has prominent characteristics in NO_2_^−^ accumulation, and its relative abundance decreased across the enrichment [64] as the accumulated NO_2_^−^ was removed through the denitrification and anammox. We detected clear enrichment of anammox bacteria (i.e., *Candidatus* Jettenia), which similar to the finding in engineered ecosystems [14, 65]. *Candidatus* Jettenia was able to become dominant at low N loading rates. Fe (II) addition also could significantly enhanced the growth rate of *Candidatus* Jettenia. We provided a lower concentration of NO_2_^−^, NH_4_^+^ and Fe (II), thus *Candidatus* Jettenia were successfully enriched. The relative high abundance of denitrifying bacteria also could provide a growth environment for *Candidatus* Jettenia.

The co-occurrence networks of species and their functions can reveal interspecific interactions [28], we found that the denitrifying and anammox bacteria formed a positive co-occurrence networks and reflected facilitative interactions. Previous study also found that high environmental stress resulted in more positive connections than competitive interactions [66]. The coupled denitrification and anammox across this enrichment may be partly due to their interactions allowing them to coexist and occupy similar niches [57]. Moreover, the network stability of denitrifying and anammox microbial communities was formed to resist normal environmental stress [67].

Recent studies indicated that different taxonomic microorganisms might perform similar functions, but functionally similar bacteria generally could not coexist unless niche differentiation exists or environmental conditions temporally changed [4]. However, it remains unknown for long-term co-evolution of complex communities [67]. We found a good correlation between anammox and denitrification during this enrichment. Generally, different microorganisms have different metabolic strategies to survive [46]. Although anammox in this enrichment ecosystem might be regulated by the metabolites of denitrifying bacteria, which also could use diverse metabolisms to regulate other biochemical processes [68]. Synergistic interactions among different populations formed across the anammox enrichment were critical to maintain the ecosystem stability [69]. Moreover, the high abundance of nucleotide sugar metabolism and amino sugar oxidation pathways at the EA stage suggested that anammox may degrade some unnecessary sugar to ensure the availability of amino acids for protein synthesis [70]. The exchanges of amino acids, vitamins, and other biotic co-factors also could affect the anammox and denitrifying community assembly [71]. Thus, a high abundance of amino acids metabolism and biosynthesis were also identified at the EA stage. This is consistent with previous study, which indicated that degradation of more amino acids to synthesis new proteins under high environmental stress [72]. Previous studies have also demonstrated that heterotrophs realized changes in enzyme activities and metabolic pathway, thereby providing a stronger ability to resist external stress [73]. Therefore, they have to modulate their metabolic patterns to reinstate themselves when they were subjected to the inorganic carbon sources. Furthermore, anammox bacteria have to rely on these heterotrophs coexisting active protein degraders to supply substances for anammox growth, which may partially explain the high abundance of amino acids metabolism and biosynthesis during the reactor start-up. Microbial cross-feedings have been reported in the anammox enrichment system, especially for carbon and energy sources [74]. Subsequent reports showed that the extracellular polysaccharides produced by the anammox bacteria could be used as carbon source by certain heterotrophs [75]. Here, we presented that multiple cross-feedings could exist during the reactor start-up. SDN harbored complete pathways for the biosynthesis of most amino acids, especially some with high biosynthetic cost. For instance, SDN genome could confirm the production of Valine, serine, threonine, betaine, cysteine, and Pantothenate. This was in contrast to AMX, which lacking the synthetic pathways of the above mention amino acids. These were inconsistent with the results of previous studies [76]. Therefore, it was supposed that SDN could degrade the extracellular proteins, and the resultant amino acids were likely be used either by for AMX genomes. Cross-feeding of secondary metabolites, including folate and biotin cofactor, has been reported as synthesizing them is important for many biological processes [77]. We observed that the AMX lacked the many cofactors and vitamins biosynthetic pathways, whereas the abundant SDN were enriched with these metabolic functions. THF is a necessary subunit for anammox CO_2_ fixation, MOCO is a cofactor for many oxidoreductases, which are involved in the anammox Wood − Ljungdahl pathway (WLP) [74]. The Biotin is a key gene in the pathway for fatty acid biosynthesis. These processes are a feeding, indicating the potential cooperation between AMX and SDN. At the same time, we found that most SDN carried DNRA functional genes. Denitrification and DNRA can occur simultaneously in the reactor. DNRA leads to NO_2_^−^/NO_3_^−^ reduction as NH_4_^+^, promoting NH_4_^+^ recycling, and the NH_4_^+^ could be oxidized by the *hao* gene. NH_4_^+^ loop plays a key role in the anammox process. Previous research had found that *hao*-related enzymes could also carry out nitrite reduction to NO [78]. The *hao* gene are ubiquitously detected in SDN, which was supposed to fulfill the function of NO_2_^−^ and NO generation for anammox metabolism. In summary, we concluded that the growth of AMX could be attributed to the metabolic interactions of SDN in anammox system. This study confirmed that the most dominant denitrifiers (e.g., SDN) harboring the energy metabolism, amino acid metabolism, and cofactors and vitamin biosynthetic ability to cross-feed with anammox and SDN encoding the function for a NH_4_^+^ loop, which could affect anammox activity.

## Conclusions

**Fig. 9:**
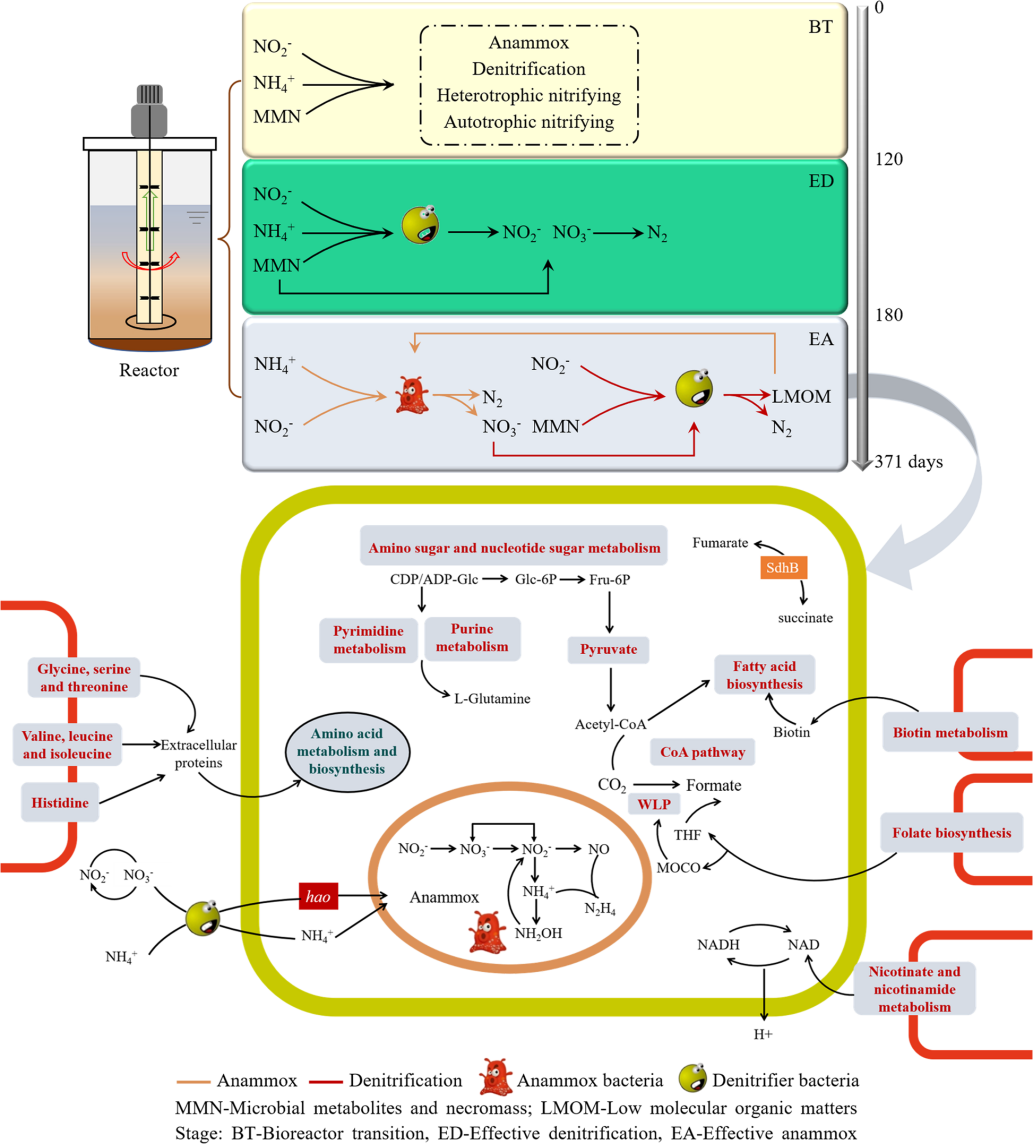
A conceptual model showing the potential coupling metabolism and nitrogen cycling pathways at different stages across the anammox enrichment with lake sediments in bioreactor systems

This study reveals the interactions and succession of enriched anammox and denitrifying bacteria across the start-up of anammox enrichment with eutrophic lake sediments, which could be divided into bioreactor transition, effective denitrification, and effective anammox stages (Fig. 9). The diversity of anammox bacteria decreased with the operation time, but the anammox *Candidatus* Jettenia increased continuously since the enrichment of 90 days. The coexistence of *nirS*-type denitrifying bacteria could enhance the enrichment of anammox bacteria. The cooperative relationships between anammox and denitrifying bacteria formed gradually across the anammox enrichment. Exchanges of energy sources between anammox and denitrifying bacteria might affect the activity and growth of the anammox bacteria by producing or degrading extracellular proteins. Additionally, the NH_4_^+^ loop is ubiquitously detected in SDN, which could provide substrates for the growth of anammox bacteria. This study greatly expands our understanding of the interactions and underlying mechanism between denitrifying and anammox bacteria during the anammox enrichment with lake sediments, which also shed new insights into N regulation for lake eutrophication control.

## Supplementary Information

**Additional file 1:** **Table S1. **Sampling sites information and physicochemical characterization for the sediment samples. **Table S2. **Influent concentrations for the anammox enrichments. **Table S3. **Corresponding programs of the used PCR primers. **Table S4.** Linear discriminant analysis (LDA) analysis revealed that the relative abundance of genera of *nirS/K*-type denitrifiers was significantly different among the three stages (LDA score > 4.0, *p* < 0.05). **Table S5.** Genome statistics information after the anammox enrichment. **Figure S1.** Scheme of the reactor used for anammox enrichment. **Figure S2.** Influent and effluent NO_3_^-^ concentrations across the start-up of anammox enrichment with lake sediments in bioreactor system. **Figure S3.** Community diversity of denitrifying and anammox bacteria across the anammox enrichment with lake sediments in bioreactor system. The boxplot showing the Shannon and Chao1 index (a), and different letters indicated significant differences (*p* < 0.05). Principal coordinates analysis (PCoA) of denitrifying and anammox communities (b). The βNTI distribution of bacterial communities at different stages (c). **Figure S4.** The relative abundance of the genera of the *hzsB*-type anammox and* nirS/K*-type denitrifiers. **Figure S5.** The relative abundance of major phylum based on the genes of 16S rRNA. **Figure S6.** A priori model identifying interrelationships among different factors. **Figure S7.** The relative abundance of the predicted nitrogen-related genes across different stages. **Figure S8.** Ratios of 16S over the sum of *nirS* and *nirK* gene copy numbers (*nirK*+*nirS*/16S)) across the anammox enrichment. **Figure S9.** The importance of functional categories determined by the random forest analysis. **Figure S10.** The correlation analysis based on the pearson for the relationships between amino acids, cofactors, vitamins and denitrifying, anammox related genes. All the asterisks denote the significance of correlations (* <0.05, ** <0.01, and *** <0.001). **Figure S11.** The relative abundance of the genes numbers on the COG class level among five bin. **Figure S12.** AMX and SDN carrying metabolism pathways (a) and N cycling genes (b). AMX and SDN statistics information are listed in Table S5.

## Data Availability

The sequencing data were deposited in the SRA database under the accession number PRJNA828340 and PRJNA906637.
