# Supplementary material for: Ecological interactions and the underlying mechanism of anammox and denitrification across the anammox enrichment with eutrophic lake sediments
Source: Microbiome. 2023 Apr 20;11:82. doi: 10.1186/s40168-023-01532-y (PMC10116762; doi:10.1186/s40168-023-01532-y)
Supplement: Supplementary file 2 — Additional file 1: Table S1. Sampling sites information and physicochemical characterization for the sediment samples. Table S2. Influent concentrations for the anammox enrichments. Table S3. Corresponding programs of the used PCR primers. Table S4. Linear discriminant analysis (LDA) analysis revealed that the relative abundance of genera of nirS/K-type denitrifiers was significantly different among the three stages (LDA score > 4.0, p < 0.05). Table S5. Genome statistics information after the anammox enrichment. Figure S1. Scheme of the reactor used for anammox enrichment. Figure S2. Influent and effluent NO3- concentrations across the start-up of anammox enrichment with lake sediments in bioreactor system. Figure S3. Community diversity of denitrifying and anammox bacteria across the anammox enrichment with lake sediments in bioreactor system. The boxplot showing the Shannon and Chao1 index (a), and different letters indicated significant differences (p < 0.05). Principal coordinates analysis (PCoA) of denitrifying and anammox communities (b). The βNTI distribution of bacterial communities at different stages (c). Figure S4. The relative abundance of the genera of the hzsB-type anammox and nirS/K-type denitrifiers. Figure S5. The relative abundance of major phylum based on the genes of 16S rRNA. Figure S6. A priori model identifying interrelationships among different factors. Figure S7. The relative abundance of the predicted nitrogen-related genes across different stages. Figure S8. Ratios of 16S over the sum of nirS and nirK gene copy numbers (nirK+nirS/16S)) across the anammox enrichment. Figure S9. The importance of functional categories determined by the random forest analysis. Figure S10. The correlation analysis based on the pearson for the relationships between amino acids, cofactors, vitamins and denitrifying, anammox related genes. All the asterisks denote the significance of correlations (* <0.05, ** <0.01, and *** <0.001). Figure S11. The relative abun [file 40168_2023_1532_MOESM1_ESM.docx]

**Ecological interactions and the underlying mechanism of anammox and denitrification across the anammox enrichment with eutrophic lake sediments**

Dandan Zhang^a^, Huang Yu^a^, Yuchun Yang^a^, Fei Liu^a^, Mingyue Li^a^, Jie Huang^b^, Yuhe Yu^b^, Cheng Wang^a^, Feng Jiang^a^, Zhili He^a^ and Qingyun Yan^a,^*

^a^ Environmental Microbiomics Research Center, School of Environmental Science and Engineering, School of Ecology, Southern Marine Science and Engineering Guangdong Laboratory (Zhuhai), State Key Laboratory for Biocontrol, Sun Yat-sen University, Guangzhou 510006, China

^b^ Key Laboratory of Aquatic Biodiversity and Conservation of Chinese Academy of Sciences, Institute of Hydrobiology, Chinese Academy of Sciences, Wuhan 430072, China

⁎Corresponding author.

E-mail address: yanqingyun@sml-zhuhai.cn

**Table S1** Sampling sites information and physicochemical characterization for the sediment samples.

| Sample sites | Longitude | Latitude | NH_4_^+^  (mg·N/kg) | NO_2_^-^  (mg·N/kg) | NO_3_^-^ (mg·N/kg) | TN (mg/g) |
| --- | --- | --- | --- | --- | --- | --- |
| S1 | E114°21′7′′ | N30°32′53′′ | 66.81±53.91^a^ | 9.51±2.98^a^ | 7.28±0.97 ^a^ | 3.50±0.05^a^ |
| S2 | E114°22′20′′ | N30°32′17′′ | 41.91±24.68^a^ | 12.43±6.85^a^ | 2.61±1.11^b^ | 3.40±0.49^a^ |
| S3 | E114°23′34′′ | N30°32′51′′ | 33.74±27.62^a^ | 2.65±0.53^a^ | 3.06±1.97^b^ | 5.30±0.34^a^ |
| S4 | E114°23′46′′ | N30°35′31′′ | 57.74±14.60^a^ | 6.66±5.04^a^ | 2.46±0.32^b^ | 4.00±0.32^a^ |

The data presented as mean ± standard deviation (n = 4). The different superscript letters mean statistical significance (*p* < 0.05).

**Table S2** Influent concentrations for the anammox enrichments

| Time (days) | NO_2_^-^ (mg·N/L) | NH_4_^+^ (mg·N/L) | HRT (h) |
| --- | --- | --- | --- |
| 1-120 | 64.30 | 60.12 | 48 |
| 121-180 | 59.90 | 58.59 | 24 |
| 181-371 | 37.97 | 32.97 | 24 |

HRT: hydraulic retention time

**Table S3** Corresponding programs of the used PCR primers.

| Gene | Primers | Sequence (5’-3’) | Thermal profile |
| --- | --- | --- | --- |
| *nirS* | cd3aF | GTSAACGTSAAGGARACSGG | 95℃ for 3 min; then 35 cycles of 95℃ for 30s, 56℃ for 30s, 72℃ for 40s; 72°C for 5 min. |
|  | R3cdR | GASTTCGGRTGSGTCTTGA |  |
| *nirK* | 1aCuF | ATCATGGTSCTGCCGCG |  |
|  | R3CuR | GCCTCGATCAGRTTGTGGTT |  |
| *hzsB* | 396F | ARGGHTGGGGHAGYTGGAAG |  |
|  | 742R | GTYCCHACRTCATGVGTCTG |  |
| 16S rRNA gene | 338F | ACTCCTACGGGAGGCAGCAG | 95℃ for 5 min; then 30 cycles of 95℃ for 30s, 53℃ for 45s, 72℃ for 60s; 72°C for 5 min. |
|  | 806R | GGACTACHVGGGTWTCTAAT |  |

**Table S4** Linear discriminant analysis (LDA) analysis revealed that the relative abundance of genera of *nirS/K*-type denitrifiers was significantly different among the three stages (LDA score > 4.0, *p* < 0.05).

|  | Stage | LDA_value | *p*_value | Genera |
| --- | --- | --- | --- | --- |
| *nirK*-type denitrifiers | BT | 5.21 | 0.02 | unclassified |
|  | ED | 3.75 | 0.02 | unclassified |
|  |  | 3.67 | 0.02 | *Ensifer* |
|  |  | 4.42 | 0.01 | *Bosea* |
|  |  | 3.88 | 0.02 | *Sphingomonas* |
|  |  | 4.04 | 0.03 | *Sinorhizobium* |
|  |  | 4.38 | 0.03 | *Bradyrhizobium* |
|  |  | 4.79 | 0.02 | *Rhizobium* |
|  | EA | 3.86 | 0.05 | *Herminiimonas* |
|  |  | 3.51 | 0.03 | *Terriglobus* |
|  |  | 3.29 | 0.03 | *Pseudomonas* |
|  |  | 4.52 | 0.04 | *Paracoccus* |
|  |  | 3.58 | 0.01 | *Nitrosomonas* |
|  |  | 3.53 | 0.03 | *Thioalkalivibrio* |
|  |  | 4.11 | 0.02 | *Mesorhizobium* |
|  |  | 3.30 | 0.02 | *Ochrobactrum* |
|  |  | 4.12 | 0.03 | *Halopiger* |
|  |  | 3.58 | 0.04 | *Turneriella* |
|  |  | 3.33 | 0.03 | *Nitrosopumilus* |
| *nirS*-type denitrifiers | BT | 4.11 | 0.01 | *Dechloromonas* |
|  |  | 5.23 | 0.02 | unclassified |
|  |  | 4.01 | 0.01 | unclassified |
|  |  | 4.18 | 0.02 | *Cupriavidus* |
|  | ED | 4.35 | 0.02 | *Thiobacillus* |
|  |  | 4.04 | 0.02 | *Rhodobacter* |
|  |  | 4.03 | 0.01 | *Azoarcus* |
|  |  | 4.17 | 0.02 | *Pseudomonas* |
|  |  | 5.11 | 0.01 | *Thauera* |

BT: Bioreactor transition stage; ED: Effective denitrification stage; EA: Effective anammox stage

**Table S5** Genome statistics information after the anammox enrichment.

| Name | Bin Id | Genome size(bp) | Longest contig(bp) | N50 (contigs)(bp) | Mean contig length(bp) | Completeness(%) | Contamination(%) |
| --- | --- | --- | --- | --- | --- | --- | --- |
| SDN | bin173 | 3194069 | 52013 | 9940 | 6479 | 93.67 | 4.95 |
|  | bin11 | 3652005 | 109862 | 28430 | 18538 | 93.64 | 0.91 |
|  | bin40 | 3395903 | 61450 | 17572 | 9758 | 93.26 | 0 |
|  | bin1 | 4437543 | 37976 | 7709 | 5283 | 93.18 | 8.91 |
| AMX | bin97 | 2017939 | 78054 | 41148 | 29676 | 54.95 | 0 |

| Bin Id | classification |
| --- | --- |
| bin173 | d__Bacteria;p__Zixibacteria;c__MSB-5A5;o__DSPP01 |
| bin11 | d__Bacteria;p__Chloroflexota;c__Anaerolineae;o__Anaerolineales;f__EnvOPS12;g__OLB14;s__OLB14 sp900696595 |
| bin40 | d__Bacteria;p__Elusimicrobiota;c__Elusimicrobia;o__UBA1565;f__UBA9628 |
| bin1 | d__Bacteria;p__Chloroflexota;c__Anaerolineae;o__Anaerolineales;f__JABWBV01;g__JABWBV01 |
| bin97 | d__Bacteria;p__Planctomycetota;c__Brocadiae;o__Brocadiales;f__Brocadiaceae;g__Jettenia;s__Jettenia caeni |


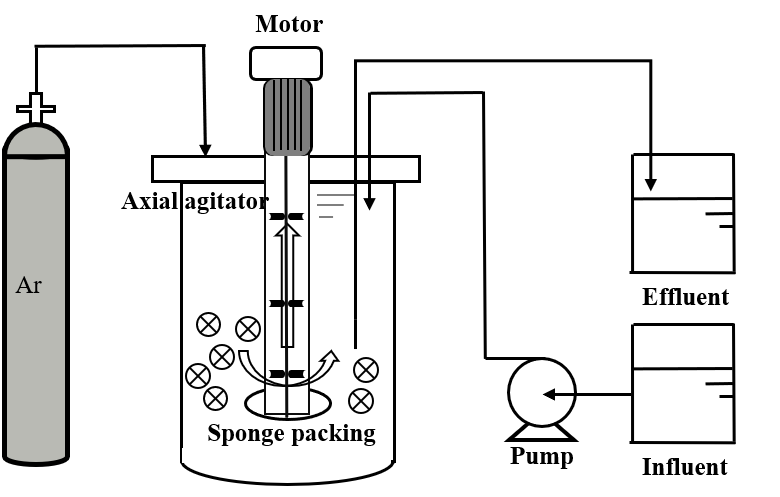


**
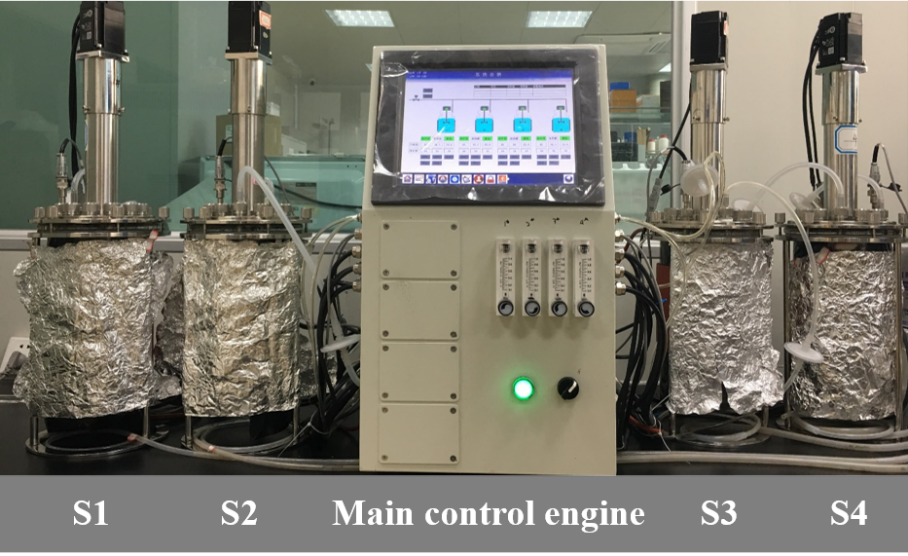
Fig. S1** Scheme of the reactor used for anammox enrichment.


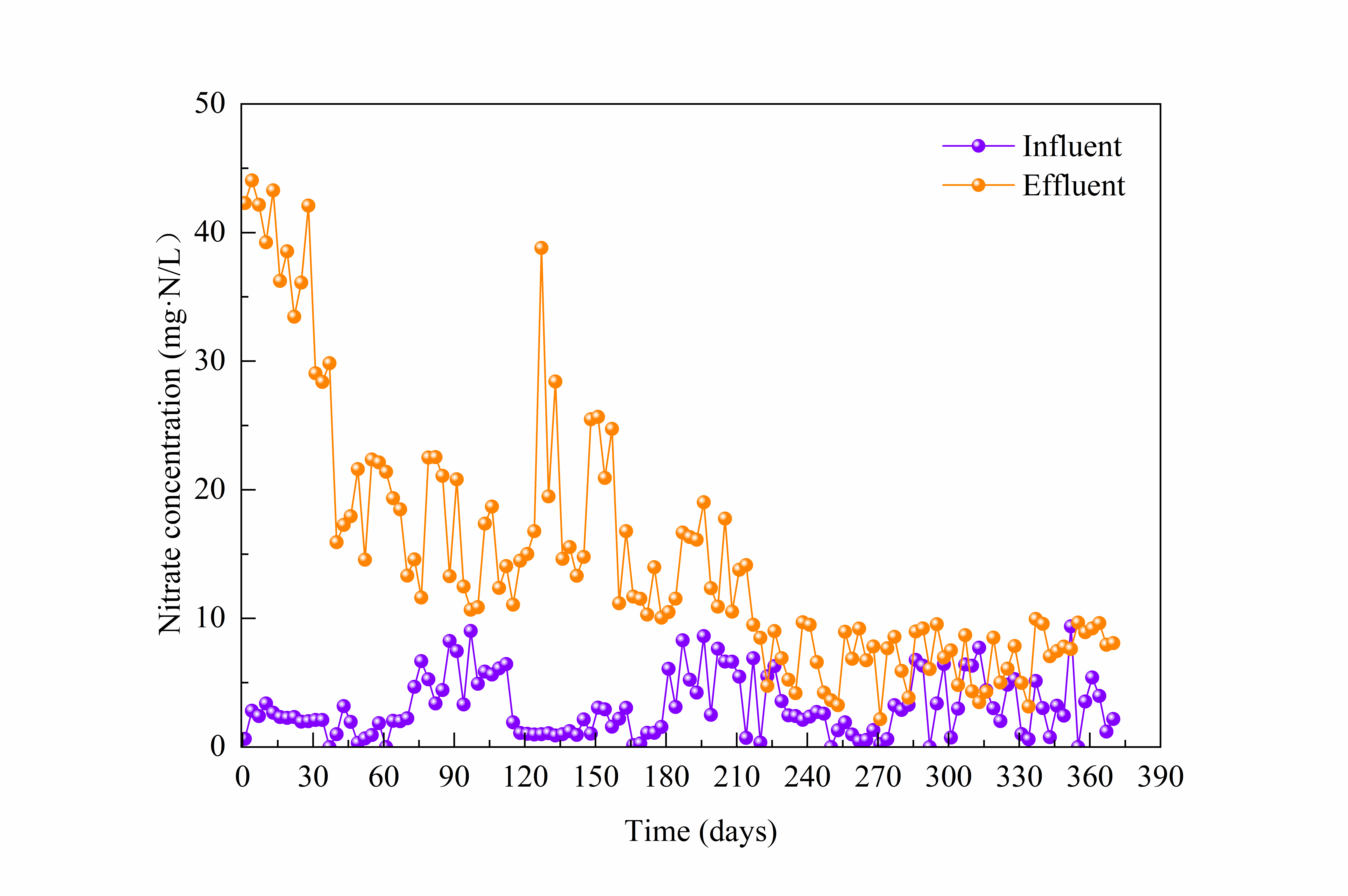
**Fig. S2** Influent and effluent NO_3_^-^ concentrations across the start-up of anammox enrichment with lake sediments in bioreactor system.


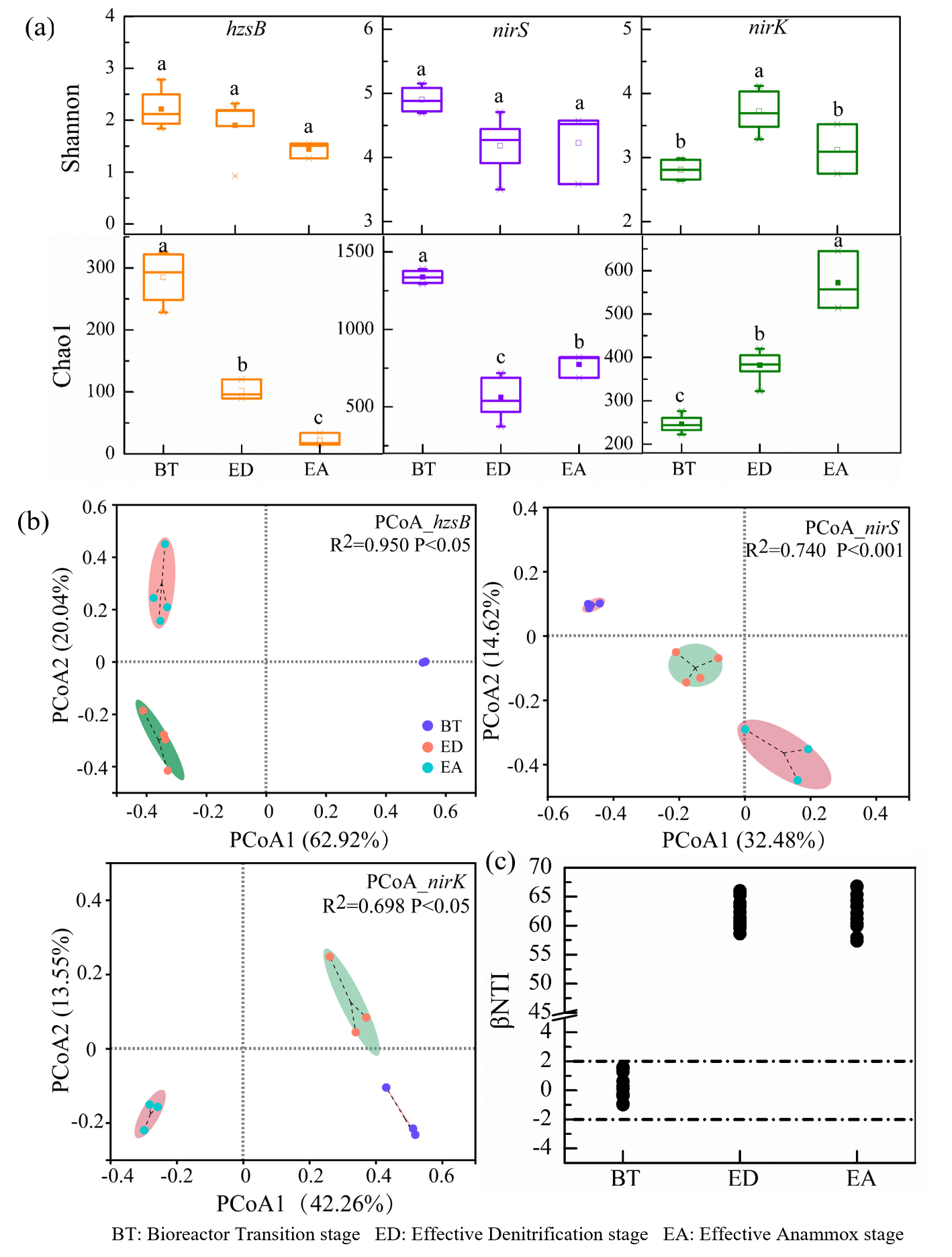


**Fig. S3** Community diversity of denitrifying and anammox bacteria across the anammox enrichment with lake sediments in bioreactor system. The boxplot showing the Shannon and Chao1 index (a), and different letters indicated significant differences (*p* < 0.05). Principal coordinates analysis (PCoA) of denitrifying and anammox communities (b). The βNTI distribution of bacterial communities at different stages (c).


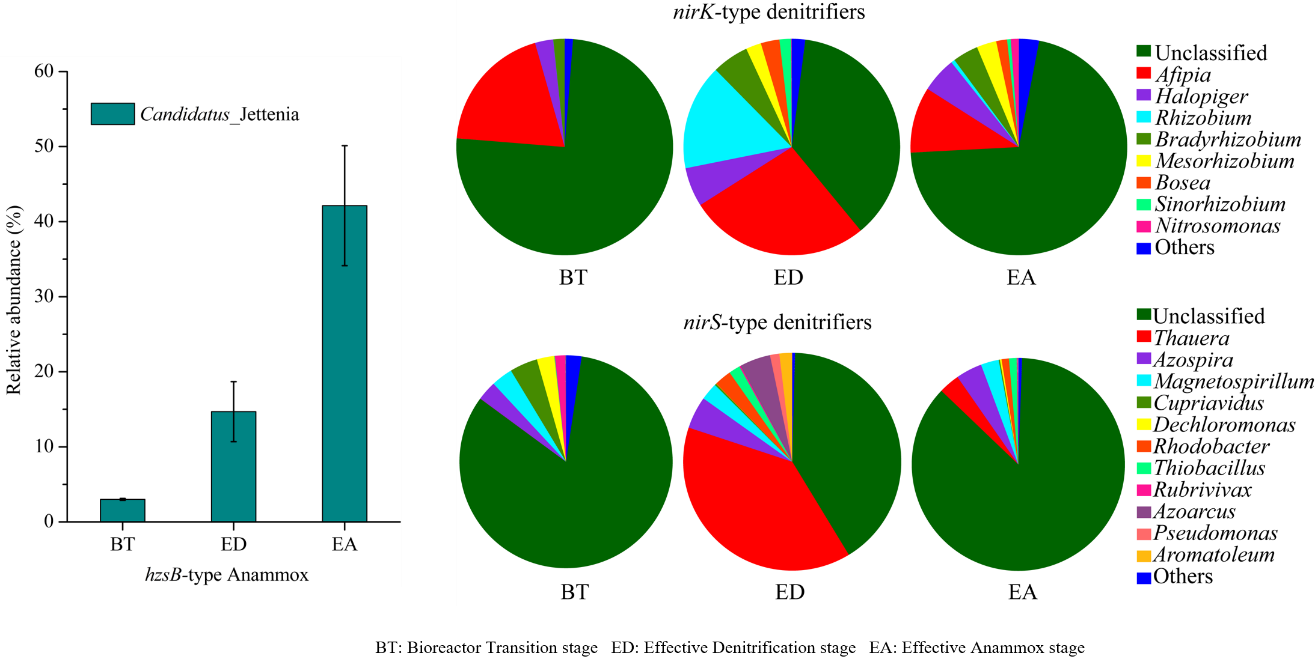


**Fig. S4** The relative abundance of the genera of the *hzsB*-type anammox and *nirS/K*-type denitrifiers


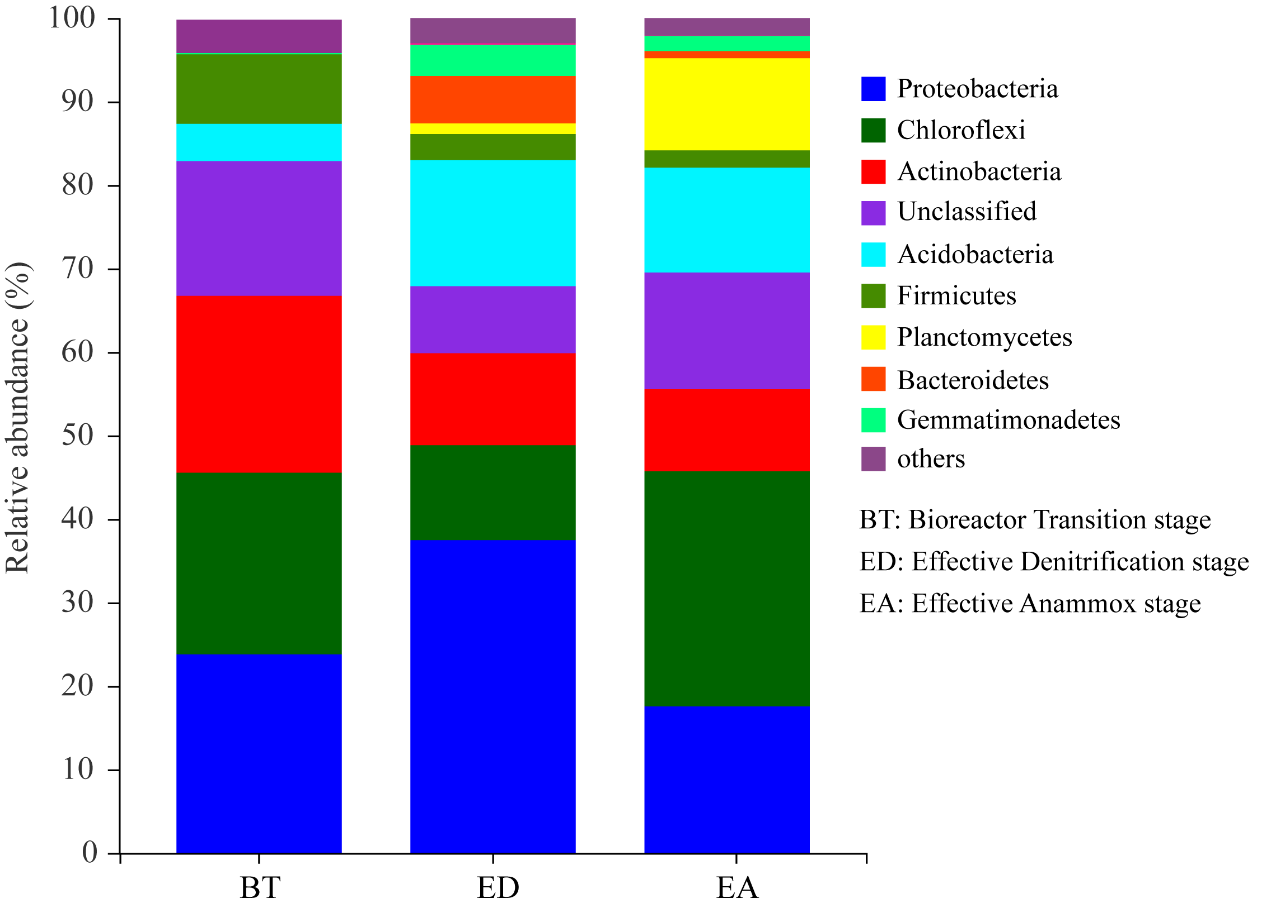


**Fig. S5** The relative abundance of major phylum based on the genes of 16S rRNA.


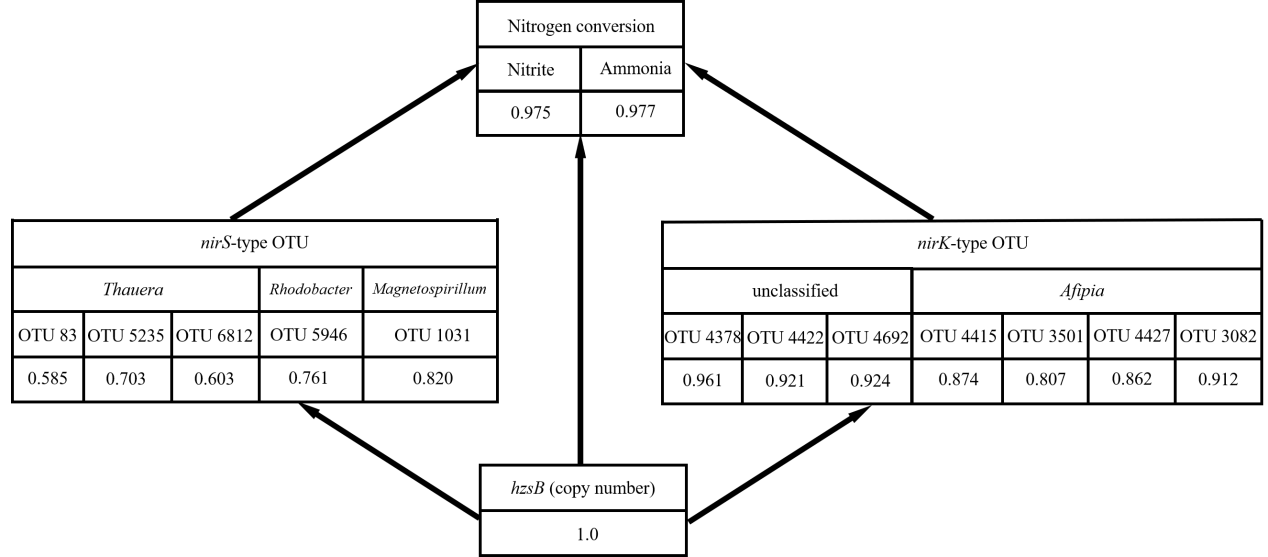
**Fig. S6** A priori model identifying interrelationships among different factors.


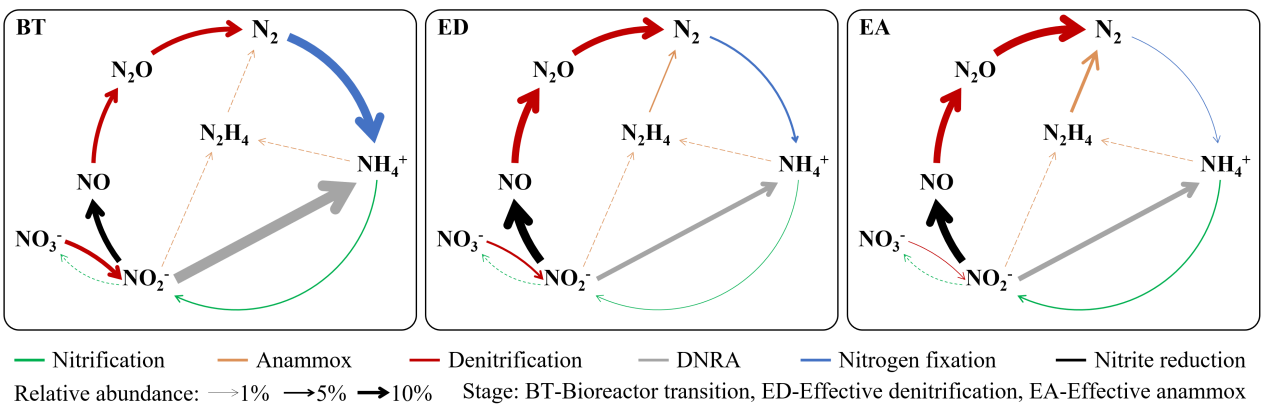
 **Fig. S7** The relative abundance of the predicted nitrogen-related genes across different stages.



**Fig. S8** Ratios of 16S over the sum of *nirS* and *nirK* gene copy numbers (*nirK*+*nirS*/16S)) across the anammox enrichment.


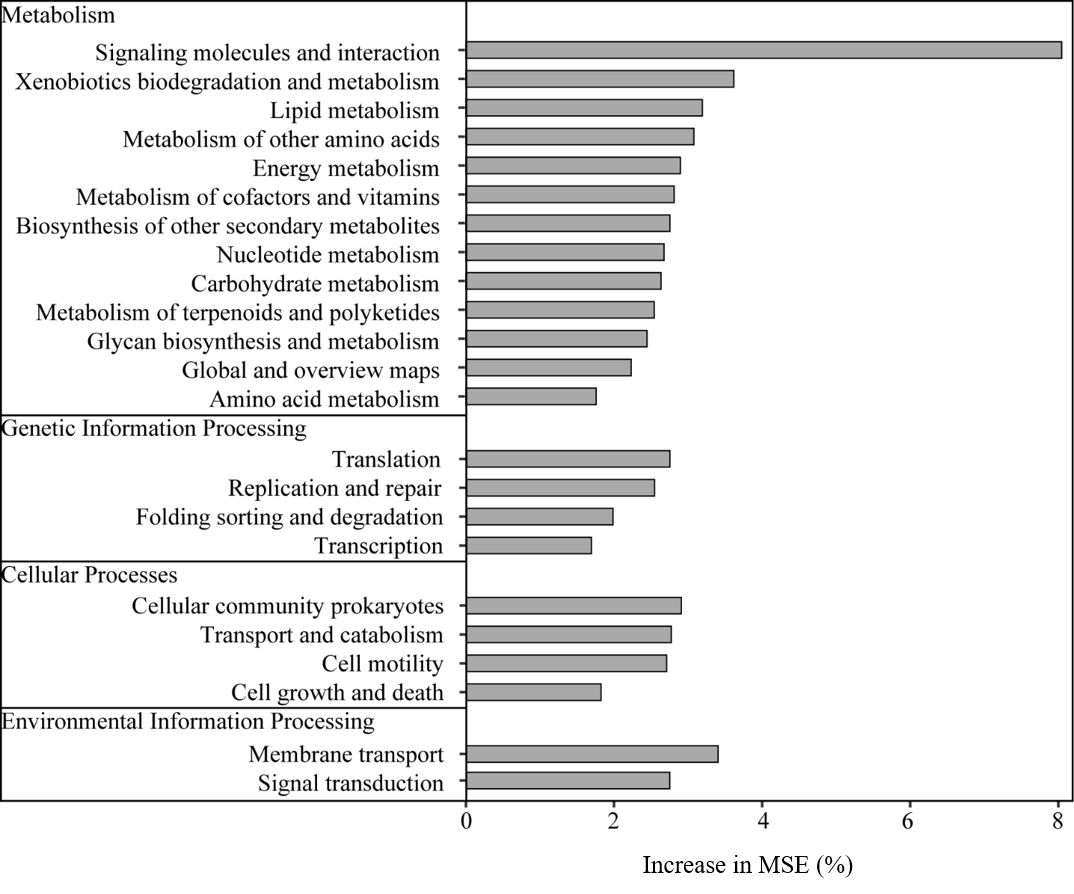


**Fig. S9** The importance of functional categories determined by the random forest analysis.


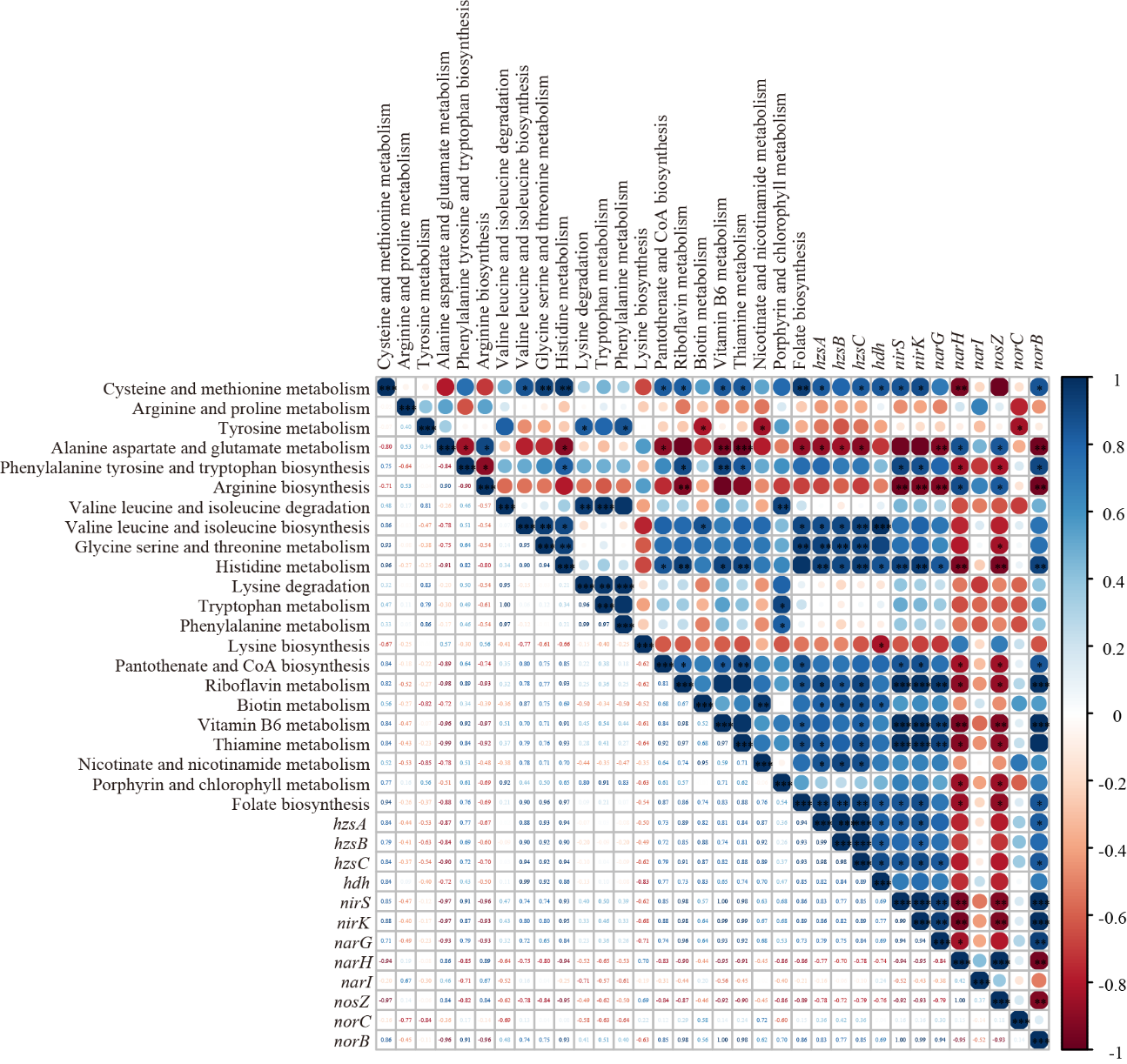
 **Fig. S10** The correlation analysis based on the pearson for the relationships between amino acids, cofactors, vitamins and denitrifying, anammox related genes. All the asterisks denote the significance of correlations (* <0.05, ** <0.01, and *** <0.001).


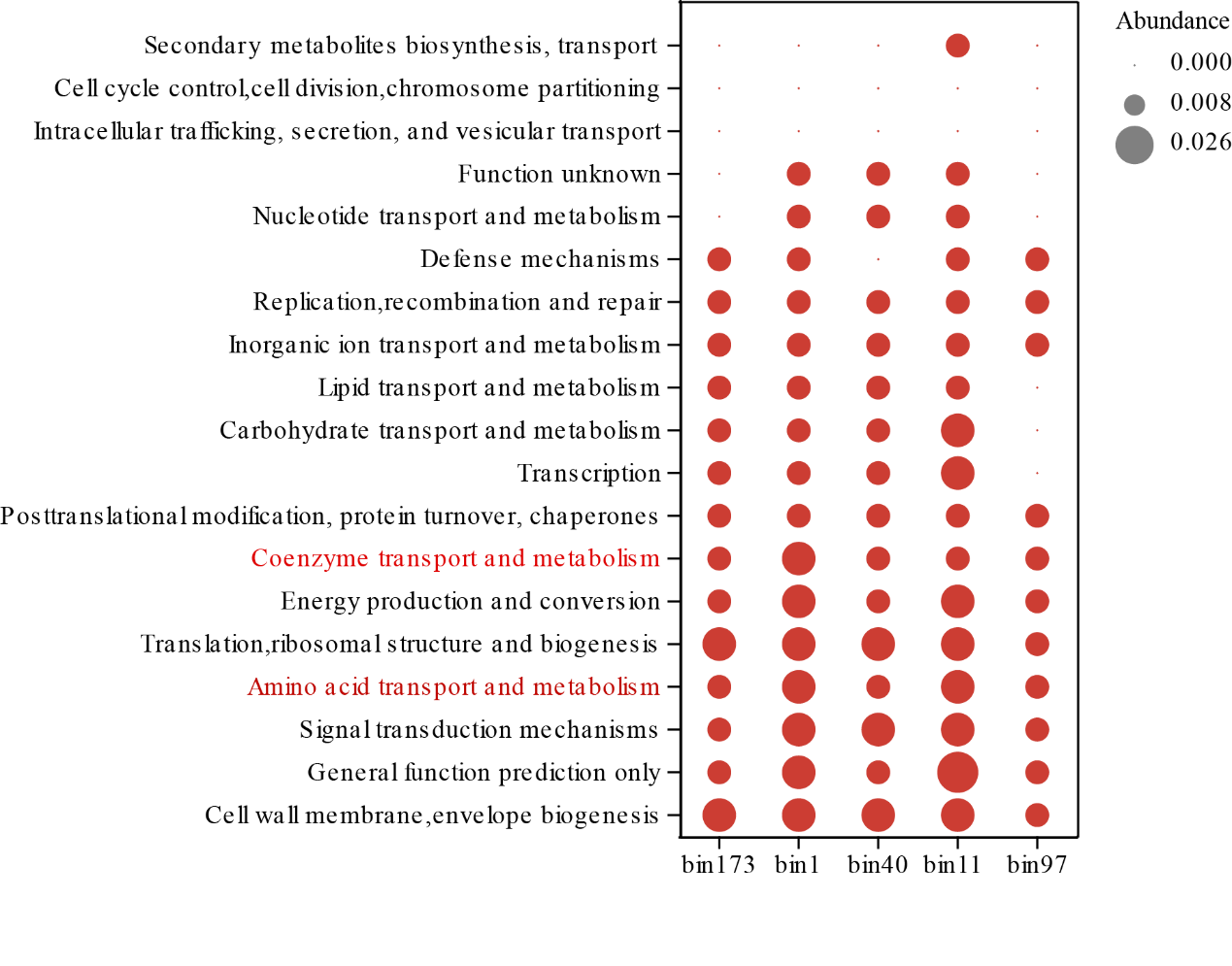


**Fig. S11** The relative abundance of the genes numbers on the COG class level among five bin.


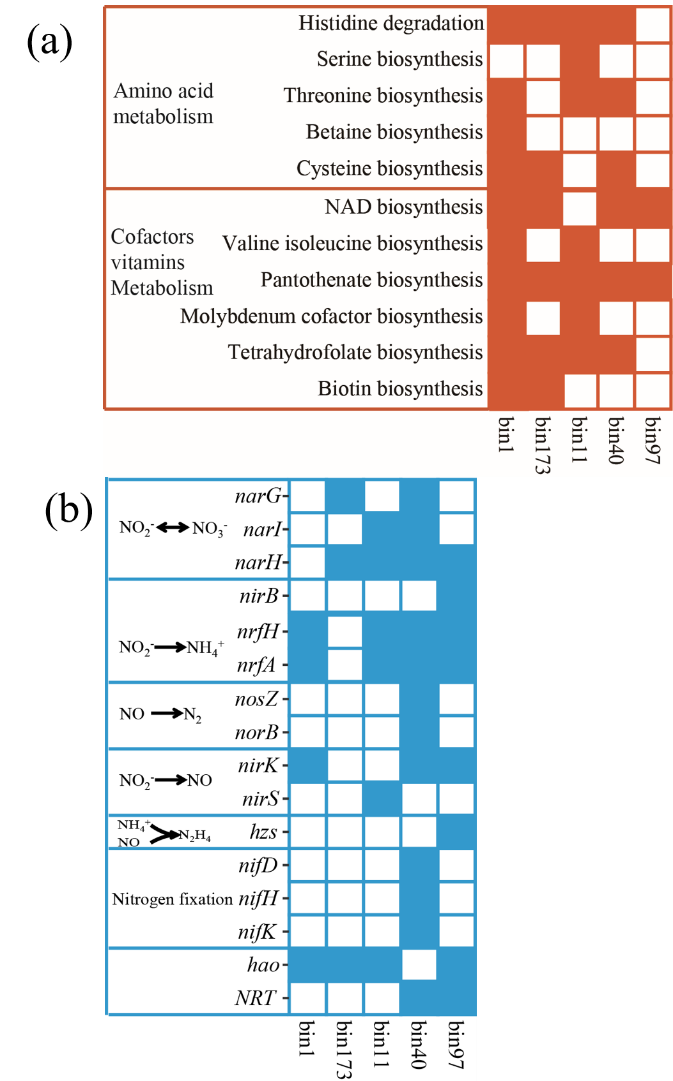
**Fig. S12** AMX and SDN carrying metabolism pathways (a) and N cycling genes (b). AMX and SDN statistics information are listed in Table S5.
